# Comparative Analysis of Structural and Dynamical Features of Ribosome Upon Association With mRNA Reveals Potential Role of Ribosomal Proteins

**DOI:** 10.3389/fmolb.2021.654164

**Published:** 2021-08-02

**Authors:** Sneha Bheemireddy, Sankaran Sandhya, Narayanaswamy Srinivasan

**Affiliations:** Molecular Biophysics Unit, Indian Institute of Science, Bengaluru, India

**Keywords:** messenger RNA (mRNA), ribosome, dynamics, normal mode analysis, flexibility, allostery

## Abstract

Ribosomes play a critical role in maintaining cellular proteostasis. The binding of messenger RNA (mRNA) to the ribosome regulates kinetics of protein synthesis. To generate an understanding of the structural, mechanistic, and dynamical features of mRNA recognition in the ribosome, we have analysed mRNA-protein interactions through a structural comparison of the ribosomal complex in the presence and absence of mRNA. To do so, we compared the 3-Dimensional (3D) structures of components of the two assembly structures and analysed their structural differences because of mRNA binding, using elastic network models and structural network-based analysis. We observe that the head region of 30S ribosomal subunit undergoes structural displacement and subunit rearrangement to accommodate incoming mRNA. We find that these changes are observed in proteins that lie far from the mRNA-protein interface, implying allostery. Further, through perturbation response scanning, we show that the proteins S13, S19, and S20 act as universal sensors that are sensitive to changes in the inter protein network, upon binding of 30S complex with mRNA and other initiation factors. Our study highlights the significance of mRNA binding in the ribosome complex and identifies putative allosteric sites corresponding to alterations in structure and/or dynamics, in regions away from mRNA binding sites in the complex. Overall, our work provides fresh insights into mRNA association with the ribosome, highlighting changes in the interactions and dynamics of the ribosome assembly because of the binding.

## Introduction

**FIGURE 1:**
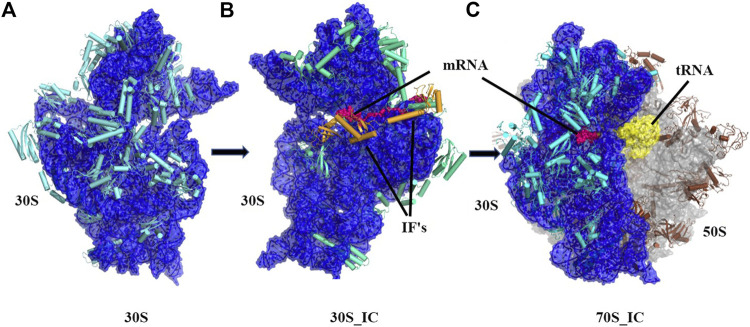
Cartoon representation showing the pathway of assembly of 70S initiation complex from 30S ribosome. **(A)** Architectural view of 30S ribosomal complex which contains 16S rRNA (blue) and 20 small ribosomal unit proteins (orange) was obtained using structure PDB ID: 4JI1, **(B)** Frontal view of 30S initiation complex, where 30S complex is bound to mRNA (dark blue) and two initiation factors, was obtained using PDB ID: 5LMN **(C)** 70S initiation complex, where 23.5S rRNA and 5S rRNA (light blue) along with 30 other proteins (grey) is shown bound to 30S complex and mRNA (dark blue).

Ribosomes are ubiquitous macromolecular complexes which help in translating genetic information from mRNA to proteins. Bacterial 70S ribosome (2.4 MDa) comprises of two subunits: a small 30S subunit that contains 16S rRNA and 20 proteins and a large 50S subunit which is made up of 23S rRNA, 5S rRNA and 30 other proteins. Various studies have, in the last two decades, described the steps involved in the assembly of the ribosome and associations between its many macromolecular components. Briefly, the pivotal work of Ramakrishnan and co-workers (Schmeing and Ramakrishnan, 2009) and several others (Wimberly et al., 2000; Gualerzi et al., 2001; Hirokawa et al., 2005; Laursen et al., 2005; Antoun et al., 2006; Simonetti et al., 2009) has led to a detailed structural analysis of the ribosomal complex at various stages of translation. Based on the analysis of several structures, it is now appreciated that in the small 30S subunit, 16S rRNA can be divided into three regions with the 5’ region representing the body, the central domain forming the platform and 3’ domain constituting the head. Likewise, the 30S ribosome complex has been divided into a head, neck, and body where the neck consists of a single helix (h44) of 16S rRNA. We now have a detailed understanding of the steps leading to 30S, 30S initiation complex (30S_IC) and 70S initiation complex (70S_IC). Briefly, translation initiation begins with the assembly of components of the 30S complex. This is followed by the formation of 30S initiation complex where 30S is bound to mRNA, initiation factors 1, 2, 3 (IF 1, 2, 3) and an initiator tRNA (Figure 1). Initiation factors kinetically regulate the initiation of translation. IF3 acts as an anti-association factor and prevents premature association of the 50S subunit, whereas IF1 sterically blocks tRNA binding with the A-site and enhances the activity of both IF2 and IF3. Initiation factors also play a role in the binding of tRNA at the P-site of the ribosome and help in improving the efficiency of translation initiation. Finally, the 50S subunit along with other tRNA associates with the 30S initiation complex resulting in a 70S initiation complex, where IFs dissociate to accommodate incoming tRNAs

In translation initiation, one of the first and significant steps is binding of mRNA. Several studies (Gualerzi and Pon, 1990; Shatsky et al., 1991; Frank et al., 1995; Greuer et al., 1999; Yusupova et al., 2001; Takyar et al., 2005; Kurkcuoglu et al., 2008) have focused on various aspects of this event and have provided key structural insights. It is now well appreciated that 30S complex associates with IFs and then enables the binding of mRNA. mRNA binds with 30S at one place and passes through 30S resulting in the formation of a tunnel. The mRNA tunnel is wrapped around the neck of the 30S subunit, with the opening between head and shoulder of the 30S forming the entry site and the opening at the platform forming the exit site. Since mRNA is required to remain single stranded for translation, proteins S3, S4, and S5 of the smaller subunit form a structure which encircles the entering mRNA and aides in its unwinding. It has been demonstrated that the ribosomal contacts with mRNA are insensitive to the mRNA sequence and involve the mRNA backbone rather than bases. At the upstream end of the tunnel, mRNA interacts with S11 and S18 such that the N-terminal of S18 interacts with 5’ mRNA. The N-terminal tail and a following loop of S11 are also involved in interactions with mRNA. At the downstream end of the tunnel, mRNA interacts with a β-hairpin of S7 with a region that also contains E-codon. Along with these interactions, mRNA also interacts with a β-hairpin loop of S12. It mainly interacts with the sidechains of basic amino acids like Arginine, Lysine and Histidine in these proteins. The interface between 30S and 50S primarily involves rRNA. S12 is the only subunit that lies at the decoding site and is involved in interactions with 50S. All the other 30S proteins that interact with 50S are known to lie at the periphery of the 30S complex.

The movement of mRNA and tRNA is associated with a “head swivel” mechanism (Wimberly et al., 2000; Ramakrishnan and Moore, 2001; Ratje et al., 2010) that involves the rotation of the 30S head domain. Translocation is known to be facilitated by the internal motions of the ribosome that include rotation of the head and body domains of the 30S ribosome (Laursen et al., 2005). mRNA translocation was shown to be coupled with the rotation of the head (Guo and Noller, 2012) and it was demonstrated that helicase activity might depend on this head movement (Zhang et al., 2009). It was also reported that the presence of IFs and tRNA induces the rotation of head and that this movement might be essential for the binding of 50S subunit (Julián et al., 2011). Further, it has been suggested that the tilting of the head of 30S is owing to its association with tRNA (Nguyen and Whitford, 2016). Indeed, it has been proposed that mRNA translocation is driven by tRNA movement (Ling and Ermolenko, 2016).

There have been many attempts to obtain a detailed map of the ribosome function. A wide range of computational techniques like explicit-solvent calculations, molecular dynamics simulations and elastic network models (ENM) have been employed to study various types of motions of ribosome. So far, explicit solvent simulations were used to predict the diffusion coefficient of t-RNA inside the ribosome (Whitford, Onuchic, and Sanbonmatsu, 2010) and to understand peptidyl transfer mechanisms (Åqvist et al., 2012; Makarov et al., 2016; Bock et al., 2018). Few studies have reported translocation events which include head and body rotation (Nguyen and Whitford, 2016), P/E hybrid formation events (including the effect of SSU rotation on tRNA motion (Levi et al., 2020), or rotation events of the small subunit (Levi et al., 2017). Molecular dynamics (MD), in combination with quantum mechanical model calculations, would provide higher accuracy in understanding transitions and reaction rates. Classical mechanical models can be combined with explicit-solvent methods to predict the free energies of the ribosome and its interactions. These methods have been employed to study free-energy changes of small RNA and are being continuously refined to provide more accurate description of RNA dynamics. To complement the knowledge from these calculations, coarse-grained elastic network models can describe the collective and correlated motions of the ribosome (Bahar et al., 2010). For large systems like ribosome, it is very difficult to perform all atom simulations because of its huge demand for computational resources and difficulties in interpretation of cooperative motions. Elastic network models (ENMs) such as Gaussian network model (GNM) and Anisotropic network models (ANM) that explore the relation between function and dynamics have been shown to be useful to study global motions (Wang et al., 2004). Typically, these methods have been validated by comparing with crystallographic temperature factors (Park et al., 2013). The low frequency motions obtained using these elastic network models are often associated with functionally important motions (Case, 1994; Cheng et al., 2006; Bauer et al., 2019). Given the complexity of the system, employing various models will reveal a wide range of perspectives to understand the ribosome.

Coarse grained elastic network models have been widely used to understand various aspects of the motion and dynamics in ribosome complexes. These studies have shed light on the rachet like motion of the ribosomes that play a significant role in translocation of mRNA and tRNA (Wang et al., 2004; Wang and Jernigan, 2005; Kurkcuoglu et al., 2009). Such motions are widely facilitated by the mobile elements of L1 and L12 from the larger 50S subunit (Zimmermann et al., 2016). Dynamics of the ribosomes were studied to understand the allostery between decoding center and peptidyl transfer center (Guzel and Kurkcuoglu, 2017).

Allostery is known to play a significant role in signal transmission in this gigantic molecular machine (Guzel and Kurkcuoglu, 2017). There are two classical models (Monod et al., 1965; Koshland et al., 1966) on allostery that consider perturbation of a residue at one site induces a conformational transition between multiple conformations that aid in biological function. Change (typically binding) of the effector alters the protein conformation using single or multiple pathways. Studying allostery is very significant in understanding the function of a macromolecule. There are many experimental techniques available that help in revealing potential allosteric pathways, but these are extensive and time consuming. In recent years, there has been a significant development in computational methods to study allostery (Chennubhotla and Bahar, 2006; Atilgan et al., 2007; Sethi et al., 2009; Long and Brüschweiler, 2011; Erman, 2013; Di Paola and Giuliani, 2015; Proctor et al., 2015; Srivastava and Sinha, 2017). One of the most popular *in-silico* techniques is the structural network analysis based approach. Network approach helps us to study the changes in structure at various sites, on the basis of various network parameters. Variation in these parameters, when compared between complexes, enables a detailed analysis of structural differences. Network approach can be used to identify most significant nodes of a network and shortest paths between effector and sensor residues (del Sol et al., 2006). Many previous reports have used network approach to study various paths of information flow in ribosome. One such study addressed the significance of the ribosomal shape using the network models of ribosome (Kurkcuoglu et al., 2008). With years of research to understand ribosome functionality, there remain many unexplored regions that require attention. One such area is the understanding of significance of mRNA binding and the role of the smaller ribosomal proteins. Our study aims to provide fresh and novel insights into the ribosomal complex formation in the light of smaller subunit proteins and mRNA dynamics. We employ ENM and protein network approach to address this open question.

In this study, we examine the conformational changes induced in the ribosome by the binding of mRNA and other initiation factors. For this, we compared the structures of the ribosomal complex in three main structural contexts: 30S ribosome complex (without mRNA), 30S initiation complex (30S complex bound with mRNA and Initiation factors 1 and 3) and 70S initiation complex - 30S complex bound to 50S complex containing mRNA and other tRNA. To determine the extent of structural change in the ribosomal complex during its transition from the 30S to 70S_IC complex, we calculated global and local RMSD values of proteins constituting the complex. Such analysis is also useful to analyze the conformational changes in the 30S initiation complex because of 50S interaction in the 70S ribosomal complex. To determine the effects of the binding of 30S complex with mRNA and other initiation factors, we performed perturbation response scanning (PRS) on each protein of the complexes. Our analysis reveals an intricate coupling between several proteins that are consistently sensitive to such perturbations revealing their importance in maintaining the structural integrity of the complex. Finally, we perform normal mode analysis on the complexes and their constituents to recognize the changes in fluctuations at the residue level, which facilitate the transition of the complexes from 30S to 70S. Through our studies, we attempt to explain the mechanism and effects of mRNA binding on the 30S complex and its significance in driving the formation of 70S initiation complex. We anticipate that these findings will be relevant and applicable to prokaryotes since the mechanism of mRNA binding is believed to be similar across these organisms, although this remains to be demonstrated and verified. Our observations strongly suggest that allostery is involved in the ribosomal function that is regulated by various ribosome binding proteins.

## Materials and Methods

### Dataset

Three structures from *Thermus thermophilus*, downloaded from the Protein databank (Berman et al., 2000), were employed for all analysis described here. These include: 1) 4JI1 30S ribosome complex (without mRNA) (Demirci et al., 2013), 2) 5LMN, 30S initiation complex (30S complex bound with mRNA and Initiation factors 1 and 3) (Hussain et al., 2016) and 3) 6QNQ, 70S initiation complex - 30S complex bound to 50S complex containing mRNA and other tRNA (Rozov et al., 2019). For the purpose of this study, we refer to these three structures as 1) 30S, 30S initiation complex (30S_IC) and 70S initiation complex (70S_IC). Missing residues in all structures were modelled using Modeller v9.14 (Šali and Blundell, 1993). Final models were energy minimised using GROMACS (Van Der Spoel et al., 2005).

### RMSD Calculations

Global RMSD calculations were carried out by comparing 30S complex with 30S_IC and 70S_IC. Local structural variations for a protein were calculated by comparing its structure in various structural contexts. Distance between corresponding Cα atoms of the protein pairs were calculated after superimposing the structures onto each other. Residues showing more than twice the standard deviation from the mean distance distribution were identified as regions showing significant structural deviation. Root mean square deviation of every complex (Global RMSD) and protein (Local RMSD) was calculated using the ProDy (Bakan et al., 2011) package and Cα-RMSD was calculated per residue using VMD (Humphrey et al., 1996). Inter-protein van der Waals energy was calculated using the ANALYSECOMPLEX module of FOLDX package (Schymkowitz et al., 2005). Angle of deviation between two proteins was calculated using an in-house code, written in python3.

### Elastic Network Model

To understand residue-level flexibility, global motions were determined using the Anisotropic Network Model-based normal mode analysis (ANM). ANM is an elastic network model (ENM) in which proteins are represented as network of nodes connected by virtual springs. The nodes are Cα atoms (for amino acids) and P atoms (for nucleotides) with a spring constant γ, that is defined for atoms within a cut-off distance. Combination of various such modes can explain the states that are accessible to the macromolecular complex near equilibrium. For a given mode, the displacement of a given node from its mean position describes the residue-level flexibility for a complex. For ENM, a Cα-Cα distance cut-off of 15Å and default value of 1 was used as spring constant. Normal modes were calculated using ProDy package (Bakan et al., 2011). Modes accounting for 80% of the variance in flexibility were considered for calculation of the square fluctuations and cross-correlation. The obtained square fluctuations were normalized and converted to Z-Score. Residues with Z-Score >2 or <−2 were considered as highly flexible.

### Perturbation Response Scanning to Identify Effector and Sensor Residues

Perturbation-response scanning (PRS) is employed to study the impact of a single residue perturbation on the whole macromolecular complex. It is based on sequential application of linear response theory (LRT), to study the origins of structural changes undergone by protein molecules. It involves systematic application of forces at singly selected residues and recordings of the linear response of the whole protein. The response is quantified as both the magnitude of the displacements undergone by the residues and their directionality. We used the PRS module of ProDy on the elastic network model of the structures in all three complexes. In the PRS module, a perturbation is applied by employing a 3*N*-dimensional force vector, based on Hooke’s law *F* = *H*⋅Δ*R*. Then, displacements of nucleotides/residues are analyzed considering the overall network, as a response to that perturbation. Each residue is perturbed one at a time, at least 1,000 times, by exerting force with random direction and unit magnitude and the response of all other residues for such perturbations are recorded. An *N* × *N* PRS matrix (heat map) is generated to display the influence and sensitivity profiles of nucleotides/residues. The *j*th column of the PRS matrix represents the response of all residues to the perturbation at residue *j*. The average of all the elements in this column points to the signal transmission potential of residue *j* as a sensor. The *i*th row of the matrix describes the response of *i*th residue to perturbations at all other sites. The average of the elements along the row indicates the potential of the residue to act either as a propagator or as an effector. The row and column averages of the resultant vectors are used to identify the effector or sensor residues (Atilgan and Atilgan, 2009). Residues that cause maximum displacement in the structure upon perturbation are termed as effector residues, while the residues that respond maximally with altered dynamics to several perturbations are termed as sensor residues. For the analysis performed here, residues with effectiveness and sensitivity values greater than two were considered as effectors/sensors.

### Network Representation of Protein Structure and Analysis

We employed network approach to understand the structural difference and to elucidate the significant communication pathways. Structures in the dataset were represented as undirected weighted graphs consisting of nodes and edges. Here, each node is located at Cα (residue) or P (nucleotide). Each node is connected to the neighbouring node by an edge such that edge lengths indicate the strength of interactions. This residue interaction network was obtained using RING software (Piovesan et al., 2016). Using network python package, we analysed and compared the residue interaction networks of mRNA bound and mRNA unbound structures based on network properties such as degree distribution, betweenness centrality and closeness centrality. Degree distribution is employed to interpret loss and gain of interactions between the compared structures. Betweenness centrality is useful to understand the significance of the residue in the context of communication among all the residues. Closeness centrality represents the extent of information transfer from one node to all the other nodes. High betweenness value implies that such residues act as a bridge in a large number of pathways while high closeness centrality values indicate that such residues are well connected and hence communicate quickly with all the other nodes. We have used top 10% of the nodes with high degree and centrality measures for the comparison.

### Communication Pathways

We have used the method where ENM was coupled with Markov stochastic model based on information theory and spectral graph method, to identify and assess signal propagation in the protein complex (Chennubhotla and Bahar, 2006; Chennubhotla and Bahar, 2007). In this method two basic quantities- hitting and commute times were calculated based on ENM fluctuations and were defined as the communication ability of the residue, to measure the “information transfer” across the network of residues in the structure. We have used the methodology obtained from Hu (2021) to calculate commute and hit values of each residue and represented them as matrix of inter-residue contacts in the structures. We have used these matrices to obtain the communication path between sensors and effectors. Cytoscape (Shannon et al., 2003) was used for visualization of network.

## Results and Discussion

### Conformational Changes in the Ribosome During Its Transition From 30S to 70S Initiation Complex

Global RMSD was computed between the identical components of the three complexes, 30S, 30S_IC and (70S_IC) to determine the extent of structural change in the ribosomal complex during its transition from the 30S to 70S_IC complex. The Global RMSD value among the three complexes was observed to be in the range of 2 to 2.4 Å implying that the binding of mRNA along with IFs or 50S does not significantly alter the global backbone conformation of the complexes. Also, large structural variations of the complex were not observed during the transition.

Given that the comparisons attempted here involve complexes with multiple protein components, we also computed the local structural deviations to determine the extent and implications of local variations at the level of individual proteins. Therefore, we calculated the structural difference or deviation of the individual proteins in the three complexes. Further, to capture the conformational changes in the macromolecular complexes resulting from changes in the orientation of proteins, we calculated the angle of deviation between the 16s rRNA and proteins of the complex. While all compared entities show some relative change with respect to each other, the proteins S3, S7, S9, S13, and S19 are found to exhibit significant local structural deviation in the range 2–3.5 Å. These changes are reflected in the angle of deviation as well (Table 1). We find that all these proteins lie in the head region of the 30S complex and are involved in the positioning of tRNA at its binding site. Interestingly, we find that the two proteins S9 and S19, although not directly involved in interaction with mRNA, exhibit significant conformational change. The distal location of S9 and S19 in the complex suggests that they may be involved in allosteric effects in the complex.

**TABLE 1:** Calculation of root mean square deviation (RMSD) and angle of deviation among proteins of 30S complex.

Protein name	30S vs. 30S_IC		30S_IC vs. 70S_IC		30S vs. 70S_IC	
	RMSD(Å)	Angle of deviation (°)	RMSD(Å)	Angle of deviation (°)	RMSD(Å)	Angle of deviation (°)
S3	2.42	4.5	1.84	1.92	3.46	5.44
S4	1.39	0.76	2.16	2.29	2.71	2.73
S5	0.795	1.11	1.23	0.92	1.016	2.02
S7	3.17	4.42	2.39	4.04	3.24	6.8
S9	3.05	3.82	2.5	2.62	3.36	5.99
S11	2.55	2.39	2.56	0.83	2.1	2.56
S12	1.39	1.28	1.4	1.16	0.03	0.12
S13	3.9	4.19	2.67	2.44	4.31	5.59
S15	1.77	1.58	2.02	2.62	0.95	3.03
S17	1.8	1.45	2.02	1.61	0.611	0.86
S18	2.31	1.04	2.65	1.44	1.52	1.06
S19	3.17	4.44	2.94	0.88	4.34	4.99

Our analysis shows that the head region of 30S undergoes structural displacement and protein rearrangement to accommodate the incoming mRNA and other proteins. Pioneering work of Ramakrishnan and coworkers and several others (Lodmell and Dahlberg, 1997; Wimberly et al., 2000; Clemons et al., 2001; Ramakrishnan and Moore, 2001; Ramakrishnan, 2002; White et al., 2014) have addressed the significance of mRNA binding with 30S subunit. Our analysis of local structural deviations involving individual proteins corroborates this finding since proteins that show considerable structural deviation lie in the head region.

### Binding of mRNA to 30S Complex Results in Changes in the Protein Interaction Network

**FIGURE 2:**
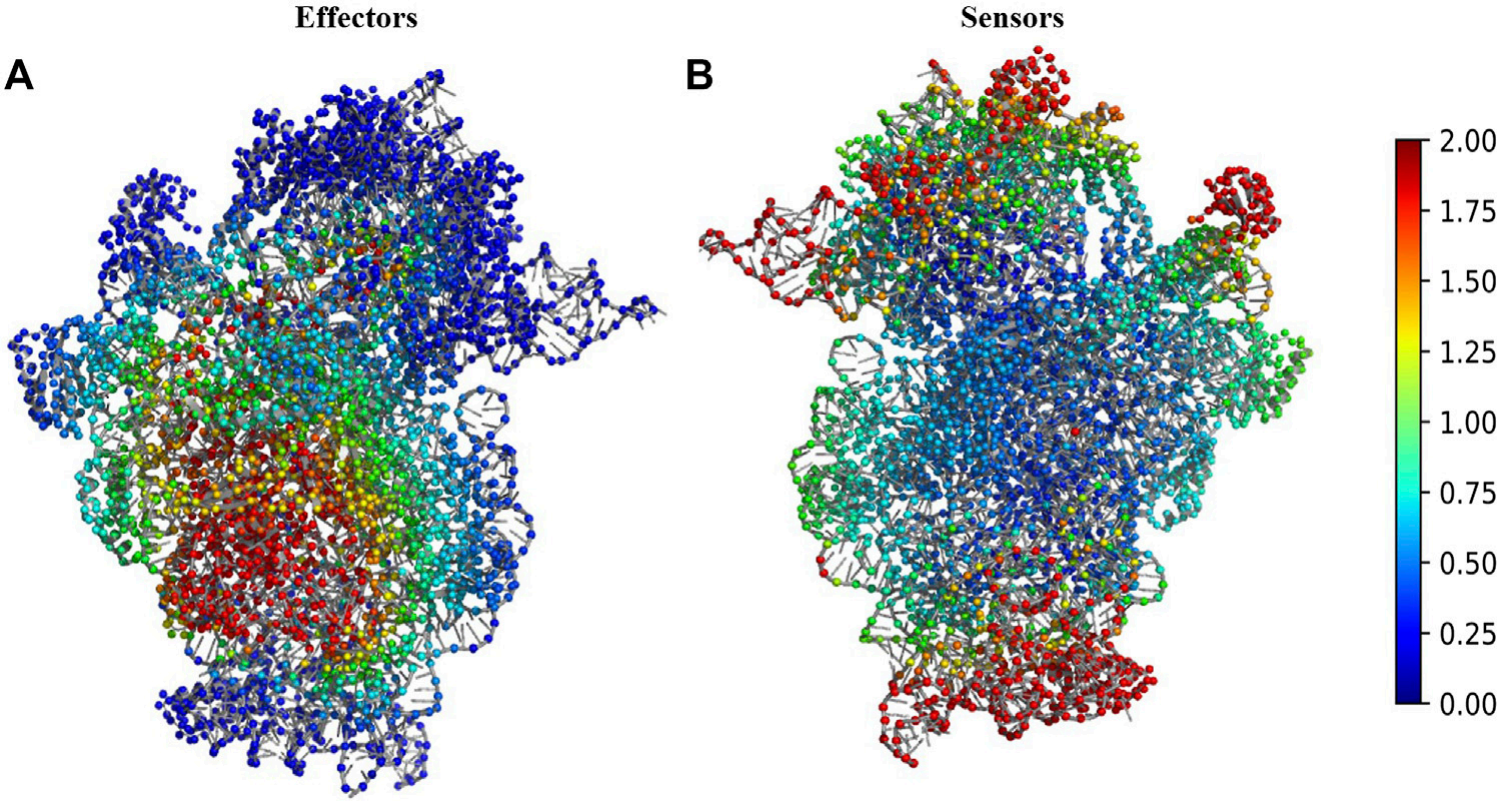
Structural map of the PRS analysis profile of 30S ribosome complex. **(A)** The structure of 30S complex is shown in cartoon. Regions were colour coded according to their effectiveness values. Residues with effectiveness greater than two are considered as effectors. Red regions depict the strongest effectors and blue regions correspond to the weakest effector sites. **(B)** The structure of 30S complex is shown in cartoon and regions were colour coded by their sensitivity values in response to perturbation. Residues with sensitivity greater than two are considered as sensors. Red regions are the susceptible sensor sites and blue regions correspond to the most insensitive sites.

We performed Perturbation response scanning (PRS) on all three complexes to determine the changes in inter protein network upon binding of 30S complex with mRNA and other initiation factors. We mapped the location of the global effector and sensor residues on the 30S complex, based on the PRS analysis profile of the 30S complex (Figure 2). We find that the global effector residues lie in a region involving residues from the proteins S5, S9, S10, S12, S16, S17 and residues from 16S rRNA. In contrast, the sensor residues were found to lie predominantly in peripheral regions of the 30S complex (Figure 2B). These include residues from S7, S9, S13, S19, S20 proteins and other 16s rRNA residues associated with them.

Comparisons of the results from PRS analysis of the complexes in various structural states reflects the inherent dynamics of the complex since, effector and sensor residues are not constant throughout the assembly process of the 70S initiation complex. The sensitive residues from 30S are represented in Supplementary Figure 1A and these are distinct from the residues of 30S_IC (Supplementary Figure 1B). Similarly, change in dynamics is also evident in effector regions of 16S rRNA in 30S and 30S_IC (Supplementary Figures 1C,D). These results suggest that the association of mRNA and IFs can alter the dynamics of 16S rRNA.

**FIGURE 3:**
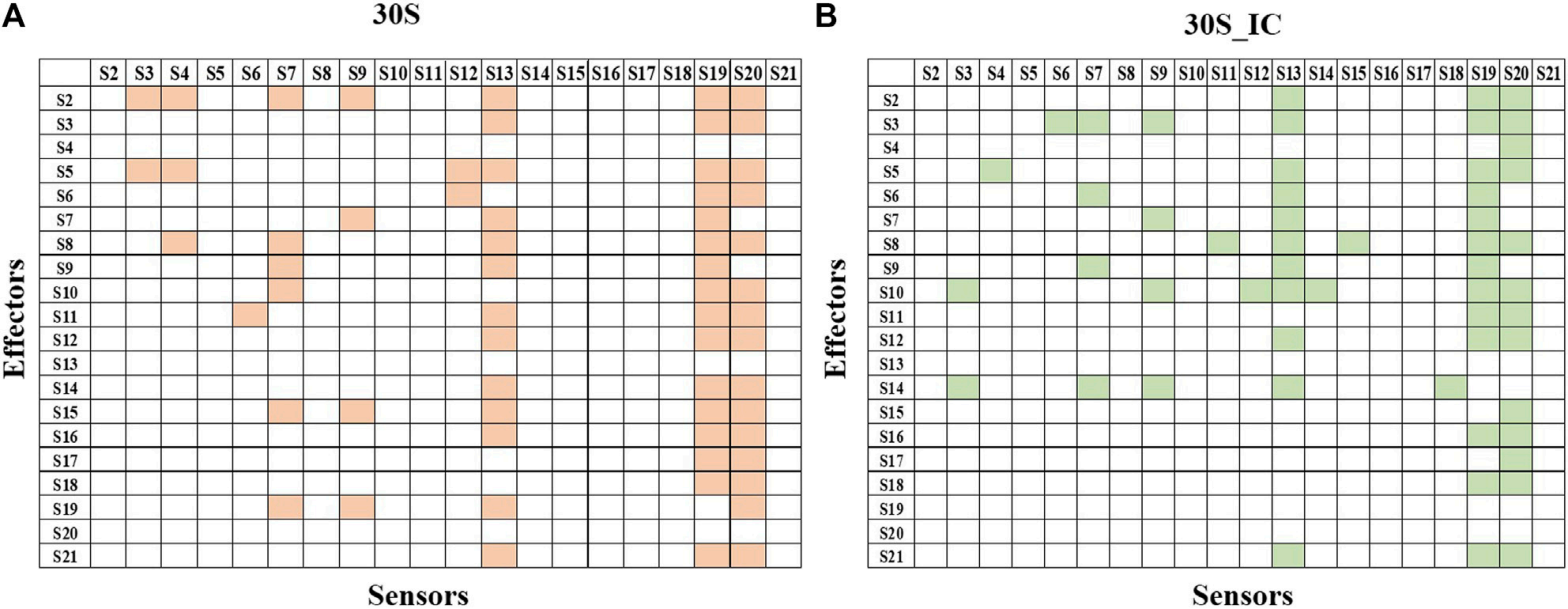
Protein-protein interaction network of 30S and 30S_IC. Protein-protein interaction network was built based on the data obtained from PRS of the entire complexes. If perturbation of a residue affects a residue from another subunit, then they were represented as interacting pair. Binding of mRNA along with IFs changes the protein interaction network of 30S complex. **(A)** Subunit interaction network of 30S complex is shown in the 2X2 matrix. Presence of interaction between any two protein components is shown in orange. Absence of interaction is indicated by a blank cell. **(B)** Subunit interaction network of 30S initiation complex is shown in the 2X2 matrix. Here, as in **(A)** presence of interaction is shown in green.

Next, we studied the effect of perturbation of each protein on the other proteins of the complex. The 2X2matrix in Figure 3 maps the effect of such perturbation between various components of the complex and is useful to understand the underlying crosstalk between proteins of the complex. From our studies, we find that S13, S19, and S20 act as universal sensors, since perturbation of any residue in the complex resulted in perturbation of these proteins (Figure 3). On examining these structures, we found that these proteins act as a bridge between the 30S and 50S in the 70S complex. We also observe that there are changes in the protein interaction network induced by mRNA and IFs. S13-depleted ribosomes are known to show translocation deficiency/are unable to undergo translocation (Cukras and Green, 2005). Likewise, ribosomes lacking S20 are defective in mRNA binding and protein association (Tobin et al., 2010). The absence of S20 was shown to result in poor assembly of the 70S complex leading to defects in the translation initiation. As shown in Figure 3, S20 interacts with all the proteins except S4, S9, and S13. These results collectively emphasize the role of individual proteins and their interactions in maintaining the structural integrity of the complex. Further, all the ribosomal proteins are important in terms of rRNA architectural support without which, the complex RNA-dependent protein synthesis machinery would become functionally suboptimal.

### Structural Fluctuations in 30S During Assembly of the Ribosomal Complex

The association of a macromolecule with an existing complex could produce a structural change or alter the dynamics of the complex or affect both. To investigate if this is observed in 30S, we calculated and compared the structural differences and fluctuations of proteins in 30S, 30S_IC, and 70S_IC, using Cα-deviation and normal mode analysis.

To cross-validate the ENM models that were used and add confidence to our results that are based on fluctuations involving the Cα-trace, we performed all atom-level and Cα-level ENM calculations on one of the protein components of the complex. Also, we performed ENM calculations on other available mRNA bound and unbound ribosome structures with better resolution than the structures analyzed in this study, to assess the influence of resolution of structures on the motions obtained using NMA. Consistency of results in both searches showed that the ENM models do not face the risk of over interpretation since they employ fluctuations involving the Cα-trace (details in Supporting Information 1.1.1, 1.2.1, 1.2.2 and Supplementary Figures 2, 3).

Further, to ensure that the fluctuations predicted by the ENM are accurate, we compared the B-factors obtained from experiments with B-factors predicted by the ENM models. Details of the methodology and results are available in supplementary data (Supporting Information 1.1.2, 1.2.3 and Supplementary Figure 4).

#### Messenger RNA Entry Gate Proteins Guide the Binding of Messenger RNA

**FIGURE 4:**
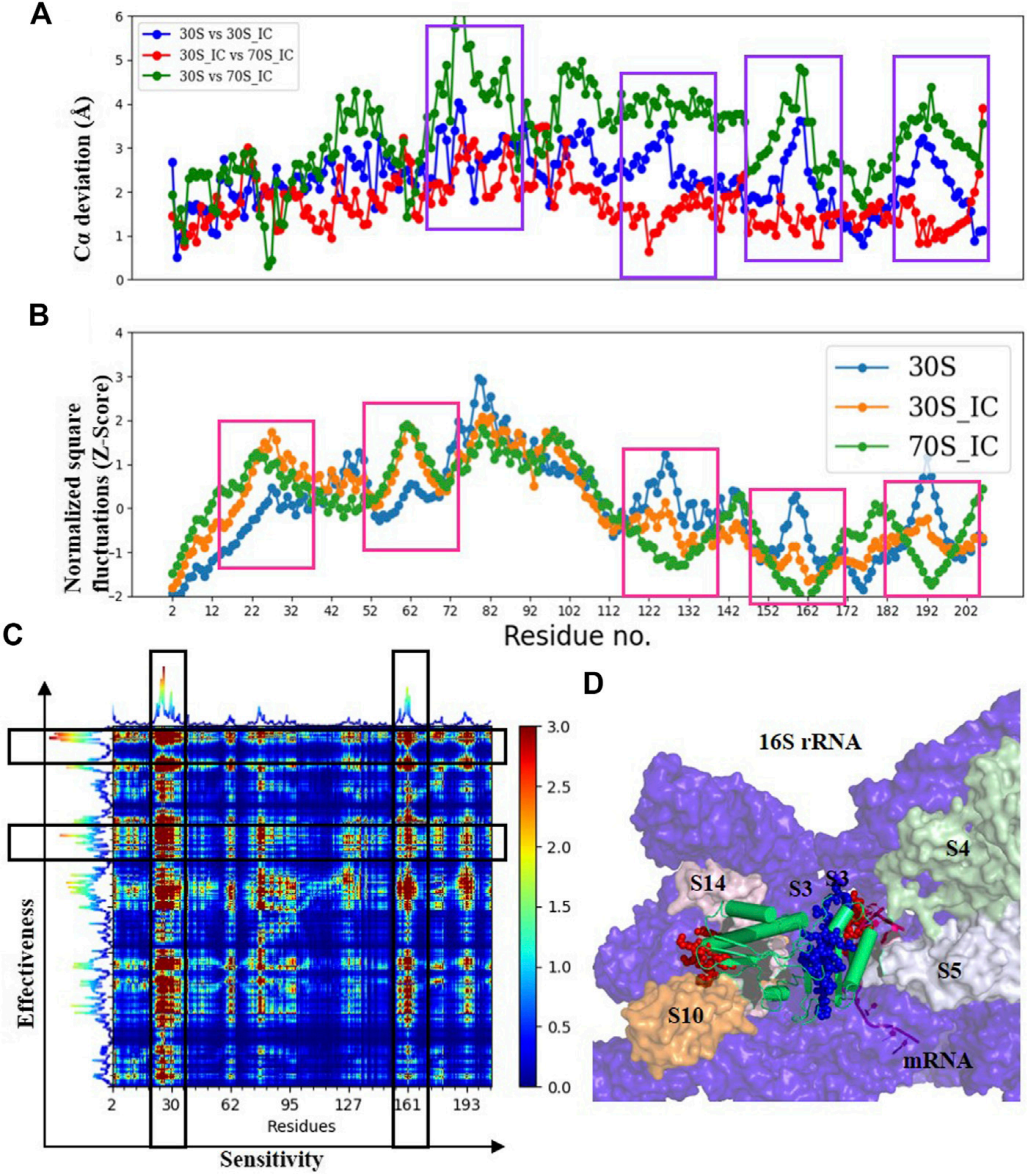
Understanding the dynamics of S3 **(A)** Cα-deviation plot for S3 in pair wise comparison of its deviation in all the complexes is shown in a scatter plot. Higher deviation indicates higher local structural variability among those structures. The regions displaying higher local structural differences have been marked with dark blue rectangles. **(B)** Scatter plot of normalized square fluctuations of S3 from 30S, 30S_IC and 70S_IC (in the form of Z-Score). Residues with Z-Score values above 2 and below −2 are considered to show higher flexibility. The regions showing significant difference in flexibility have been marked with red rectangles. **(C)** The PRS analysis of the S3 protein shown as a heat map with sensitivity profile on the X-axis and effectiveness profile on the Y-axis. Regions marked by a vertical rectangle contain most sensitive residues while horizontal rectangle depicts the most effective residues. **(D)** A region of the structural map of the 30S complex (4JIL) highlighting the S3 (yellow) interacting partners. S3 interacts with mRNA (light orange), S14 (blue), S10 (deep orange) and 16S rRNA (deep blue) in surface representation. The effectors (blue) and sensors (red) identified in S3 are represented as spheres.

Comparisons of Cα deviation of 30S with 30S_IC and 70S_IC complexes show that S3 undergoes significant structural change (Figure 4A). These structural changes are found to correlate well with changes in Z-scores (Figure 4B). When 30S was compared with 70S, we observe that the regions of S3 in 30S_IC, which lie at the interface between S10 and S14 show higher flexibility. On the other hand, the regions of S3 involved in interaction with mRNA, 16S rRNA and S5 show reduced flexibility. Further, we observe that the flexibility at the N-terminus of S3 does not change upon formation of 70S_IC, whereas, towards the C-terminus, it mirrors that of S3 in the 30S_IC (Figure 4A). When we coupled these results with the effects of PRS of individual residues, the residues of S3 involved in interactions with S14 and S10 again emerge as sensitive residues (Figures 4C,D), while the residues involved in interaction with mRNA behave as effector residues (Figures 4C,D). Collectively, these findings suggest that S3 undergoes significant structural and dynamics change during the transition from 30S to 70S_IC complex. Further, they also raise the possibility that S3 may act as a mediator for the flow of information from S14 and S10, which are themselves involved in tRNA interaction in the complex with mRNA and S5. It is important to note, based on the available structures that S3 lies at the bottom of the head and may interact with other proteins in the head region such as S10 and S14. It is also well positioned to interact with S5, which is located in the body of 30S complex. Earlier studies have shown that S3 along with the proteins S4 and S5 is involved in the helicase activity, where unwinding is due to the relative movement of head to the body (Takyar et al., 2005). These studies have also reported that S3 binds to mRNA in the 70S ribosome, positioning mRNA for translation initiation. Our results show the significant structural and dynamics changes of S3 and lend support to these findings.

To test the robustness of the relationships and ensure that our interpretations of long range interactions from ENM are valid and accurate, we applied an alternate residue interaction network method, to compare the changes in structural network between the mRNA bound and unbound forms. Here, network analysis of S3 shows that while the residues interacting with mRNA do not show many changes in terms of degree and betweenness centrality, changes in closeness centrality are high, implying that these residues act as effector residues (data not shown).

Similar analysis of S4 does not show significant change in all the three compared complexes, except for the region 142–162 that shows higher local structural variability. This region that is involved in interaction with the mRNA double helix (Supplementary Figure 5A) is flexible in all the three structures and is required for stable binding of S4 (Supplementary Figure 5B). In addition, our results show that residues 42–57 of S4 act as sensors and are involved in interaction with S5, while residues 87–112 act as effectors (Supplementary Figure 5C). Analysis of residue interaction networks for this protein reveals that the residues 43, 46 and 54 of S4 figure among the top 10% of the residues with highest betweenness. This shows that they are significant for the structure of the complex and that they may have important roles in maintaining network integrity. These residues are known to interact with 16S rRNA (Supplementary Figure 5D) implying, that information is transferred from rRNA to S5. This is further supported by our observation on interaction energy calculations for S4 where interaction energy values with rRNA are observed to improve upon complex formation. The same analysis shows that its interaction with S5 is stronger in 30S initiation complex (Supplementary Table 1).

S5 does not exhibit a large change in either Z-score or Cα-RMSD (Supplementary Figure 6A). Regions 62–70 of S5, involved in interaction with 16S rRNA, display higher structural deviation in 70S_IC (Supplementary Figure 6B). In contrast, regions 15–25 of S5, which are involved in the interaction with mRNA, remain flexible in all the three structures, allowing the movement of mRNA through the tunnel. Interestingly, these residues exhibit very high values of betweenness and closeness centrality, suggesting their importance. We find that this region of S5 along with its C-terminus acts as a sensor while residues 105–125 form the effector region and show significant centrality values (Supplementary Figures 6C,D). Although S5 does not have a role in helicase activity, it has been reported that it helps in maintaining the fidelity of mRNA (Kirthi et al., 2006). Our results show that the interaction energy of S5 with rRNA is higher in 30S initiation complex (Supplementary Table 1) emphasizing the significance of S5 in the formation of complex with mRNA.

#### Exit Gate Proteins Regulate mRNA Movement

The ribosomal protein S7 lies near the decoding center, helping the cross-linking of tRNA at A- and P- sites. In the 70S initiation complex, S7 is directly involved in the interaction with tRNA at A-site and with mRNA using a highly flexible β-ribbon. S7 is also one of the principal regulatory elements that controls ribosomal protein synthesis by the translational feedback mechanism (Hosaka et al., 1997). We observe that the β-ribbon undergoes significant structural change (Supplementary Figure 7A) and that this region is flexible throughout the process of complex formation (Supplementary Figure 7B), facilitating the dynamic interactions of both mRNA and tRNA. The sensors in this protein were identified at the N-terminus and include residues 74–90 (Supplementary Figure 7C) that are involved in interaction with rRNA, while the effectors are residues 57–67 and 120–130 that are solvent exposed (Supplementary Figure 7D). Our network analysis shows that the sensor residues also exhibit very high changes in closeness centrality measures, high betweenness centrality and high degree values. These residues also form hubs upon interaction with mRNA. Our analysis therefore shows that the major conformational change in the whole protein occurs during transition from the 30S to 30S_IC and that this can be attributed to the binding of tRNA. This indicates that S7 plays an important role in the 70S complex formation, specifically for tRNA binding.

S11, which shares an interface with S7 is known to help S7 in the stabilization of mRNA-tRNA association. Flexibility of various regions in S11 changes upon 30S_IC formation and are restored to original levels with the formation of the 70S complex. Likewise, dynamics is also restored to normal in 70S_IC (Supplementary Figure 8B) but they might change in the process of elongation, as S11 is known to get involved in the dissociation of tRNA at E-site (Hussain et al., 2016).Our PRS results show that the C-terminus of S11 acts as sensor (Supplementary Figure 8C) and that these residues exhibit significant changes in closeness centrality. This region is involved in interaction with mRNA, rRNA and S18. Residues 61–67 act as effectors and also show high values of closeness centrality in the complex demonstrating that S11 plays a significant role in positioning of mRNA and that it receives the information from other proteins through its highly flexible C-terminal tail.

S18 is essential for the survival of plant cells and its N-terminus is directed towards the SD helix major groove (Kaminishi et al., 2007). It has been suggested that S18 plays a role in the positioning of mRNA in *Thermus thermophilus* (Jenner et al., 2005). We observe that the C-terminal tail of S18, which interacts with mRNA is the sensor region in the protein (Supplementary Figure 9A). Residues 67–75 which interacts with S6 and rRNA act as effectors in the complex (Supplementary Figure 9B). Our analysis, therefore, shows that S18 has high flexibility in the regions where it interacts with rRNA.

#### S12 Mediates 30S Interaction With 50S Complex

S12 is found near the decoding center and interacts with IF1, S17, S8 and rRNA. S12 is important to maintain pre-translocation state (Feng et al., 2013). Its interaction with rRNA plays a role in mediating 50S binding. Further, S12 is a key component in optimization of codon recognition and tRNA selection process (Demirci et al., 2013). Our results show that S12 dynamics is altered upon interaction with 50S subunit (Supplementary Figure 10B). We find that the region on S12 where tRNA binds is initially flexible but is stabilized upon association with 50S (Supplementary Figure 10B). We also find that all other domains become stabilized upon its association with 50S except for its N-terminus. Supplementary Figure 10B shows that binding of IFs makes it less flexible whereas its association with 50S complex makes it more flexible. This protein acts as a communicator by transmitting signals from tRNA to 30S complex. S12 helps in fixing the mRNA in the 30S complex upon association of 50S. We find that the N-terminus acts as a sensor and is involved in interaction with 16S rRNA, S8, S15 and effectors (55–66, 94–104) (Supplementary Figure 10C). This is also reflected in the high closeness centrality values of residues 94–104 at the N-terminus.

#### Dynamics of 50S Binding Proteins

S13 regulates translocation of mRNA with the help of S12 (Cukras et al., 2003). Structures show that its tail penetrates the P-site in 30S complex, where it runs parallel to the anti-codon stem loop. The basic residues in its tail are known to enable the interaction with tRNA and it is directly involved in the function of 30S P-site. In our calculations, we observed that there were no significant changes in the flexibility of S13 throughout the transition from 30S to 70S_IC (Supplementary Figure 11). The region that is involved in the interaction with 50S remained flexible in all three complexes. The C-terminal tail also remained quite flexible and was accompanied by large structural deviations (Supplementary Figure 11).

S15 is one of the primary proteins that orchestrates the assembly of other ribosomal proteins S6, S11, S18 and S21 with 16S rRNA (Agalarov et al., 2000). It is required in nucleation of the central domain of 30S complex. It is also known to be involved in the feedback mechanisms inhibiting its own mRNA translation (Philippe et al., 1993). Supplementary Figure 12 shows that the regions through which S15 interacts with 16S rRNA remain flexible in all the three structures. Further, the region of its interaction with 50S also has higher flexibility without much structural variation.

S17 plays a significant role in stabilizing the tertiary structure of 16S rRNA 5’ domain (Ramaswamy and Woodson, 2009). We observed no significant structural deviations in S17 in all the three complexes (Supplementary Figure 13). The residues at the interface of S17, 16S rRNA and 50S exhibit higher flexibility even after the interaction of 30S complex with mRNA and 50S ribosome. S19 is involved in the interaction with 50S complex through its N- terminus. Comparison of its dynamics in the three complexes shows that while binding of mRNA and IFs makes it less flexible, its binding with 50S makes it more flexible (Supplementary Figure 1). Large structural deviation was observed in the residues involved in interface with 50S.

### Network Analysis Reveals Possible Communication Routes for Allosteric Regulation in 30S Subunit

**FIGURE 5:**
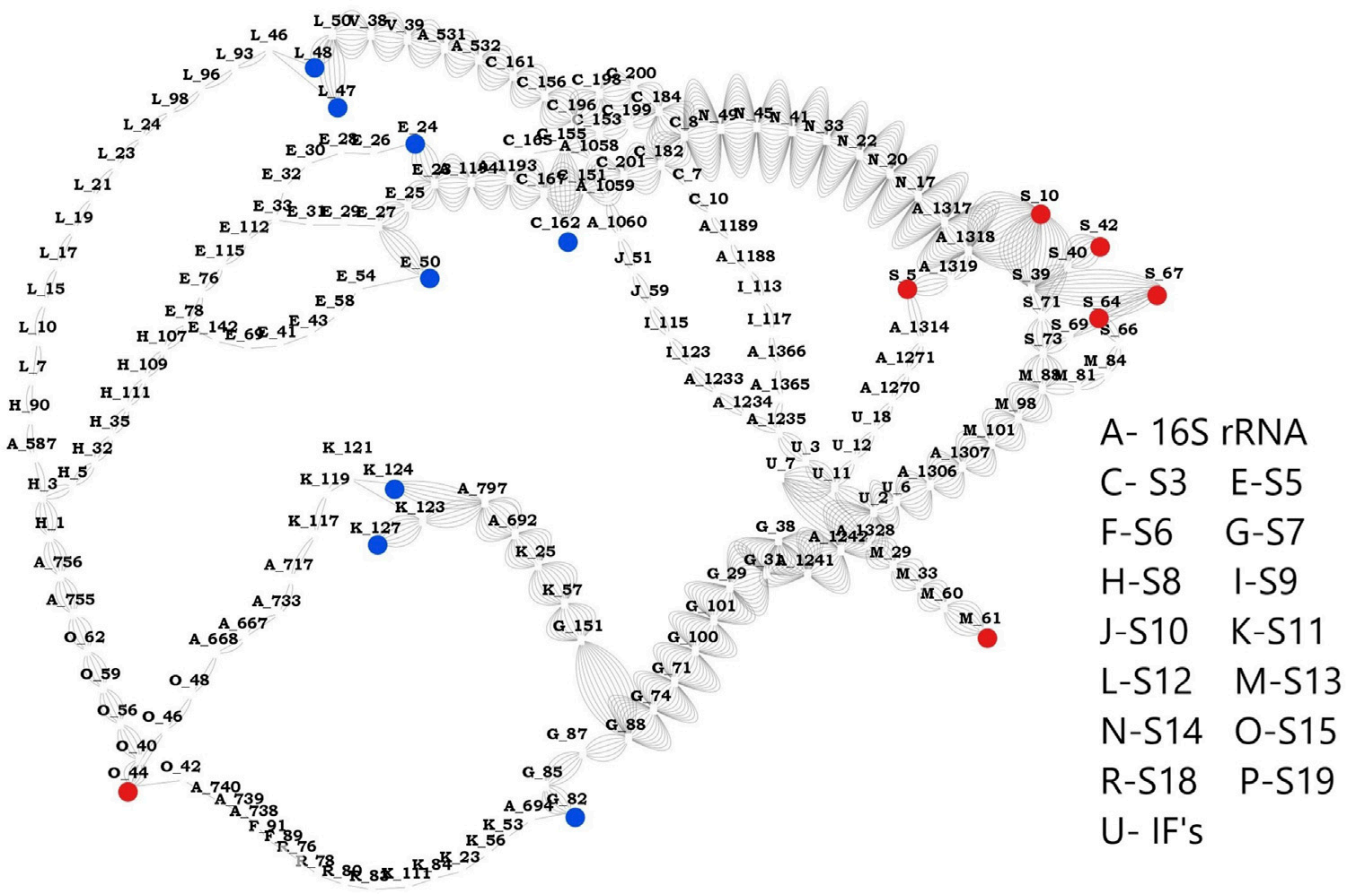
Network between sensors and effectors. This image shows the shortest path computed between the identified sensors and effectors. Sensor residues are marked in blue and effector residues in red. Protein names are represented using alphabets and their respective names are shown in the legend. Number of edges between any two nodes represents number of times that edge is crossed between sensors and effectors.

Based on the network studies and comparative analysis of the proteins in the three complexes, we discovered the most influential nodes in the proteins. Using these results, we classified the highly sensitive mRNA binding residues as source and highly effective residues as sink. We could identify 8 residues (S3:162, S5: 24, S5: 50, S7: 82, S11: 124, S11: 127, S12: 47, S12: 48) as effectors and 7 (S13: 61, S15: 44, S19: 5, S19: 10, S19: 42, S19: 64, S19: 67) residues as sensors that are connected by a total of 56 paths. Network parameters of these residues obtained from mRNA bound and unbound structures (Supplementary Figures 15–17) clearly show that mRNA association increases the total number of interactions in the complex. This leads to changes in the pathway priming it for regulation. Analysis of the pathways obtained between sensor and effector residues (Figure 5) shows that there are two dominant paths of communication between the identified effectors and sensors, one of them constituted by C-terminal residues of S3 and residues from S14. Another path involves residues from S7 and S13. S7 has been shown to crosslink the 16S rRNA and A-, P- sites both in our studies and elsewhere (Hosaka et al., 1997). Here it is shown to be play a significant role in transmitting the signal from mRNA binding residues and this likely facilitates the binding of 50S subunit. Our results provide a compelling support for allosteric linkage and communication pathways between the proteins that are also implicated in relaying information from the mRNA binding subunits to 50S binding proteins. Further experiments can be aimed at exploring these hypotheses and confirming the communication pathways between various components of the complex, through mutation/knock out studies of the above residues.

## Conclusion

Ribosome is a complex structure that is involved in protein translation and governs cellular proteostasis. Experimental methods have attempted to investigate diverse aspects of its organization and have revealed that each step is regulated by large-scale, coordinated conformational changes within the ribosome and associated translation (Noller, 2006; Korostelev et al., 2008; Zhang et al., 2009; Sheppard et al., 2010; Feng et al., 2013). Indeed, several interesting studies have shed light on its intriguing biology and examined diverse aspects of its function such as, the head swivel mechanism of 30S complex, mRNA-tRNA interactions, nascent polypeptide synthesis, role of elongation factors in the elongation step of translation, mechanics of the decoding center etc. and have furthered advancement of knowledge in these areas (Yusupova et al., 2001). Toward the goal of gaining insights into changes in the ribosomal complex during its transition from 30S to 70S_IC, which marks the start point for translation initiation, we have investigated the structural deviation of the ribosomal complex and its individual components using various complementary computational approaches.

Firstly, to obtain an overview of the extent of structural change, we computed global RMSD of the three complex structures leading to translation initiation. Our calculations show that the overall change, in terms of structural deviation of the complex, is not very significant. Such scores however, may obscure the significant local structural changes at the level of individual proteins. Therefore, we computed local RMSD between individual proteins, coupled with angle of deviation between the proteins and compared these values in the three complexes. Here, we found that several proteins undergo considerable deviation upon mRNA-binding. These include S3, S7, S9, S13, and S19, where S3 and S7 are known to interact with mRNA directly. Interestingly we find that the S9, S13, and S19, located distant from the mRNA binding site and IFs undergo considerable structural deviation, raising the possibility of their role in allosteric binding in the 70S complex. To complement our findings on structural displacement of the head region of 30S, we performed normal mode analysis of mRNA bound and unbound structures in an attempt to capture these changes. Ramakrishnan and co-workers and others have shown that the binding of IFs to 30S causes the opening of a latch at the mRNA entry site, preparing the complex for the incoming mRNA. It has been reported that a rotation movement is observed in the head after mRNA interaction, resulting in head movement such that the exit site is prepared for the mRNA, allowing it to pass through (Carter et al., 2000; Wimberly et al., 2000; Ramakrishnan, 2002). Our ENM of the ribosome complex also captures such collective motions of the complex and we observe motions similar to head opening/closure upon interaction with initiation factors and mRNA. Our results are well in agreement with the motions proposed previously (Supplementary Figure 18), lending support to these observations.

Next, we calculated residue level flexibility in individual proteins. Here, we observe that regions involved in interactions with mRNA in all the proteins, are consistently flexible throughout the transition of the complex. This finding reveals the accommodative nature of the proteins, in terms of dynamics, and suggests that this may facilitate the necessary flexibility for the movement of mRNA through the tunnel of the complex. This is further supported by results of perturbation response scanning at the level of individual proteins that identifies effectors and sensors in the complex. Here, based on our findings, we propose that S3 may act as a sensor mediating information flow to other proteins along with S4 and S5, with a role in the movement of mRNA. Comparison of these changes across the three complexes provides an opportunity to probe changes in these interactions. Our studies reveal that as the 30S complex proceeds through the transition, the roles of residues of the 16s rRNA as effectors and sensors is altered. This transitivity in their roles emphasizes their dynamic properties. Further, our PRS results of the other proteins show that S11 and S7 that interact with each other can influence binding of mRNA. This result in conjunction with the flexibility comparisons across the three complexes reveals that these proteins may have a potential role at the mRNA exit tunnel. Likely, S11 along with the help of S7 aids in the movement of mRNA and S18 helps in communication of mRNA with other proteins. Our results show that S12, the only subunit that is present close to the decoding center, is potentially involved in interactions with IFs (in 30S_IC) and 50S. Interestingly, flexibility analysis of S12 reveals that it exhibits a complete change in flexibility upon association with 50S although it does not show structural variability. We also observe changes in the dynamics of ribosomal proteins (S13, S15, S17 and S19) that are involved in interaction with 50S. Here, notably, S17 was observed to retain flexibility in its interacting residues even after its association with 50S complex.

Our interpretations on overall flexibility and dynamics of the complex are based on coarse grained models of the ribosome complex at the Cα-level. To improve confidence in our findings and to validate our findings, we checked whether our interpretations are consistent when we consider all-atom representations. For the structures analyzed in this study, all-atom representation is difficult to perform at the level of the entire ribosomal complex, therefore, we selected the S13 protein of one of the complexes. We find that the results of all-atom ENM for S13 are consistent with those of Cα-calculations (Supplementary Figure 2). Similar studies have been performed previously for other systems and cooperative dynamics of all atom and coarse-grained structures were shown to be equivalent (Doruker et al., 2002). Further, we also performed ENM calculations for other available mRNA unbound (PDB ID: 2VQE) and mRNA bound (PDB ID: 3T1Y) ribosome structures with better resolution than the structures analyzed in this study (Supplementary Figure 3). Here again, we find that motions obtained using NMA remain unchanged in various structures irrespective of their resolution values. It is important to note that in our study, we have restricted our analysis to low frequency movements and gross large-scale motions and limited our interpretation of results to fluctuations involving the Cα-trace. We have, intentionally, not extended our results to side-chain atoms. Given that our results are at the Cα level, we believe that they will remain valid even when all-atom representations become feasible for the structures analyzed here.

The robustness and validation of our findings using ENM are best tested through the applications of other models. We corroborated our findings using a residue network-based analysis. Specifically, we investigated changes in the residue interaction network of the mRNA bound and unbound structures. Such comparison allowed us to identify source and sink in the allosteric communication pathway. Our analysis suggests that there are two dominant pathways of communication, which involve proteins S3, S7, S13, and S14 that are also recognized as crucial for information flow through ENM based dynamics studies. Although our findings support the view of allosteric linkage and recognize communication pathways between the proteins that relay information from mRNA binding to 50S binding proteins, we believe that further confirmation from experiments would be necessary to demonstrate this effectively. Increase of the structural repertoire and advancements in computational techniques would open opportunities for a detailed analysis of the mRNA binding and other functions of the ribosome in future.

We have employed methods that rely on different principles to describe the collective changes in the ribosomal complex during its transition from 30S to 70S_IC and motions of proteins in this complex. On the one hand, detailed structural analysis based on the available static structures of the complexes provides an overview of regions in the complex, that undergo structural change as a consequence of transition of the complex from 30S to 70S_IC. On the other hand, we probed for regions that show collective motions in the complex using normal mode analysis. More detailed analysis of such motions helps to narrow down on residue level flexibility in individual proteins. Correlations of such flexibility with the physical location of residues in the context of the complex lends support to several earlier reported findings on the importance of such regions in the transition of the complex. Further, when we apply perturbations to individual protein components of the complex structures, we were able to recognize regions that are sensitive and may mediate information transfer between protein components of the complex. Indeed, application of network models in our analysis not only supports the importance of specific residues in the complex, in their role as potential mediators of information transfer, but are also useful to probe for their involvement in allostery. By using several methods that concur in their overall objective, we have attempted to recognize the regions in the ribosome that are dynamic and play an important role in the diffusion of information in the system. As such, our analysis holds promise for identifying communication pathways as well as sites that show high flexibility and undergo dynamic changes. In future, probing such sites through more detailed experiments such as site-directed mutagenesis, cross-linking experiments and spectroscopic methods such as site-directed fluorescence labeling or FRET, would be useful to confirm the proposed pathways for information transfer.

## Data Availability

Publicly available datasets were analyzed in this study. This data can be found here: The PDB structure data of the proteins was downloaded from the Protein data bank (https://www.rcsb.org/) under the accession numbers 4JI1, 5LMN and 6QNQ. The original contributions presented in the study are included in the article/Supplementary Material, further inquiries can be directed to the corresponding authors.
