# Supplementary material for: Comparative Analysis of Structural and Dynamical Features of Ribosome Upon Association With mRNA Reveals Potential Role of Ribosomal Proteins
Source: Front Mol Biosci. 2021 Aug 2;8:654164. doi: 10.3389/fmolb.2021.654164 (PMC8365230; doi:10.3389/fmolb.2021.654164)
Supplement: Supplementary file 1 [file DataSheet1.PDF]

Supplementary Material

1 Supplementary Data

1.1 Methods

1.1.1 Calculation of square fluctuations

To determine the reliability of results and their dependency on the resolution of the structures , we calculated square fluctuations of the mRNA bound (PDB ID: 3T1Y (Vendeix et al. 2012)) structure of resolution 2.8Å and unbound (PDB ID: 2VQE (Kurata et al. 2008)) structure of resolution 2.5Å. Square fluctuations were calculated using calcSqFlucts function of ProDy package. All-atom and Cα-fluctuations of S13 protein were also calculated using ProDy package.

1.1.2 Calculation of b-factors

To determine the reliability of results and their dependency on the resolution of the structures, we calculated b-factors using ENM models with the help of ProDy package.

1.2 Results

1.2.1 Comparison of all-atom and Cα ENM

Since, it is difficult to perform normal mode analysis at the level of the entire ribosomal complex, we selected the S13 protein of one of the complexes. We find that the results of all-atom ENM for S13 are consistent with those of Cα-calculations (Supplementary figure S1). Similar studies have been performed previously for other systems and cooperative dynamics of all atom and coarse- grained structures were shown be equivalent (Doruker, Jernigan, and Bahar 2002).

1.2.2 Comparison of ENM models of mRNA bound and unbound structures

To show that the fluctuations predicted by ENM are accurate, we used ENM models of the mRNA bound and unbound structures to calculate the cross-correlation between motions of various residues. Here again, we find that motions obtained using NMA remain unchanged in various structures irrespective of their resolution values. Results pertaining to this analysis can be found at Supplementary figure S2.

1.2.3 Comparison of b-factors

The accurate predictions of crystalline b-factors are one of the methods to determine the reliability and replicability of ENM models. We found that predicted b-factors are in agreement with the experimental B-factors as reported previously (Kalaivani and Srinivasan 2015). In addition, the correlation value between experimental and calculated b-factors was obtained as 0.657 which implies that both the b-factors are well correlated. There is a region in the 16S rRNA (Supplementary figure 3) where the values do not correlate, a peak is observed in the calculated b-factors and this peak corresponds to the missing residues in the 16S rRNA of the mRNA structure.

2 References

Kalaivani, Raju, and Narayanaswamy Srinivasan. 2015. “A Gaussian Network Model Study Suggests That Structural Fluctuations Are Higher for Inactive States than Active States of Protein Kinases.” *Molecular BioSystems* 11 (4): 1079–95. https://doi.org/10.1039/c4mb00675e.

Kurata, Shinya, Albert Weixlbaumer, Takashi Ohtsuki, Tomomi Shimazaki, Takeshi Wada, Yohei Kirino, Kazuyuki Takai, Kimitsuna Watanabe, V. Ramakrishnan, and Tsutomu Suzuki. 2008. “Modified Uridines with C5-Methylene Substituents at the First Position of the TRNA Anticodon Stabilize U·G Wobble Pairing during Decoding.” *Journal of Biological Chemistry* 283 (27): 18801–11. https://doi.org/10.1074/jbc.M800233200.

Vendeix, Franck A.P., Frank V. Murphy IV, William A. Cantara, Grazyna Leszczyńska, Estella M. Gustilo, Brian Sproat, Andrzej Malkiewicz, and Paul F. Agris. 2012. “Human TRNALys3UUU Is Pre-Structured by Natural Modifications for Cognate and Wobble Codon Binding through Keto-Enol Tautomerism.” *Journal of Molecular Biology* 416 (4): 467–85. https://doi.org/10.1016/j.jmb.2011.12.048.

3 Supplementary Figures and Tables

3.1 Supplementary Figures

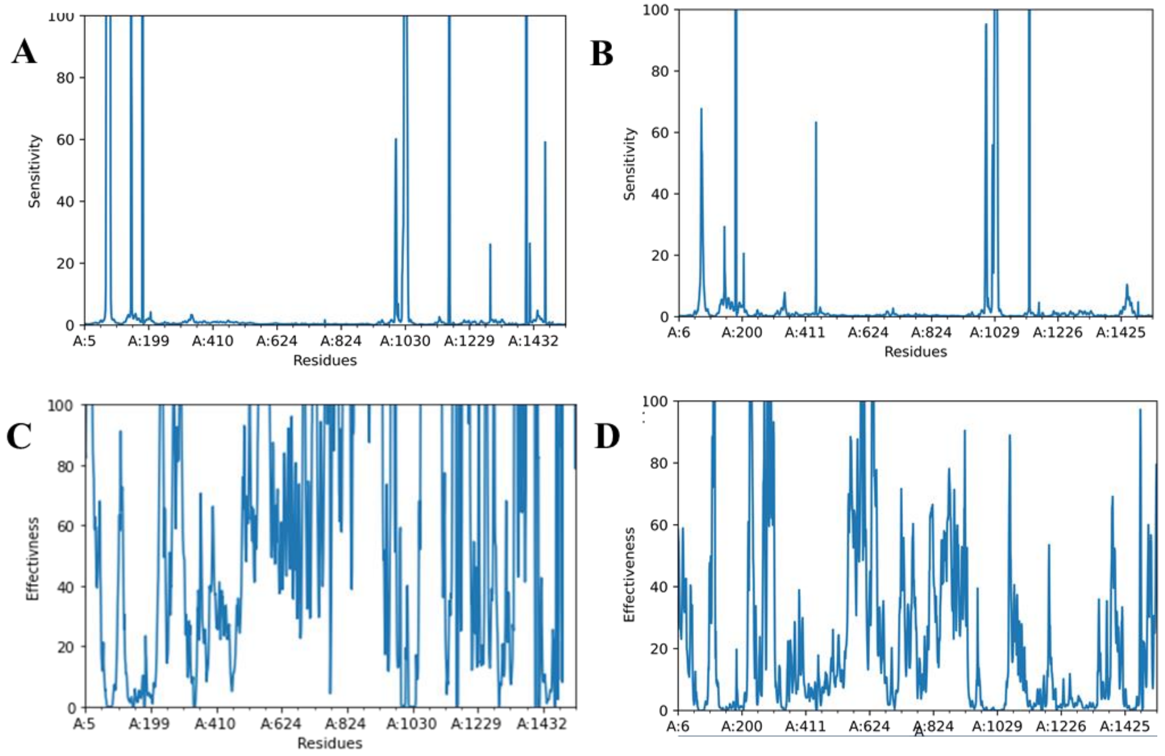

Supplementary Figure 1. Average effectiveness and sensitivity plots of 16S rRNA from 30S and 30S_IC. A and B are the average sensitivity plots of 30S and 30S_IC where Y-axis contains sensitivity values and residue number on X-axis. C and D are the average effectiveness plots with effectiveness values on Y-axis and residue number on X-axis.

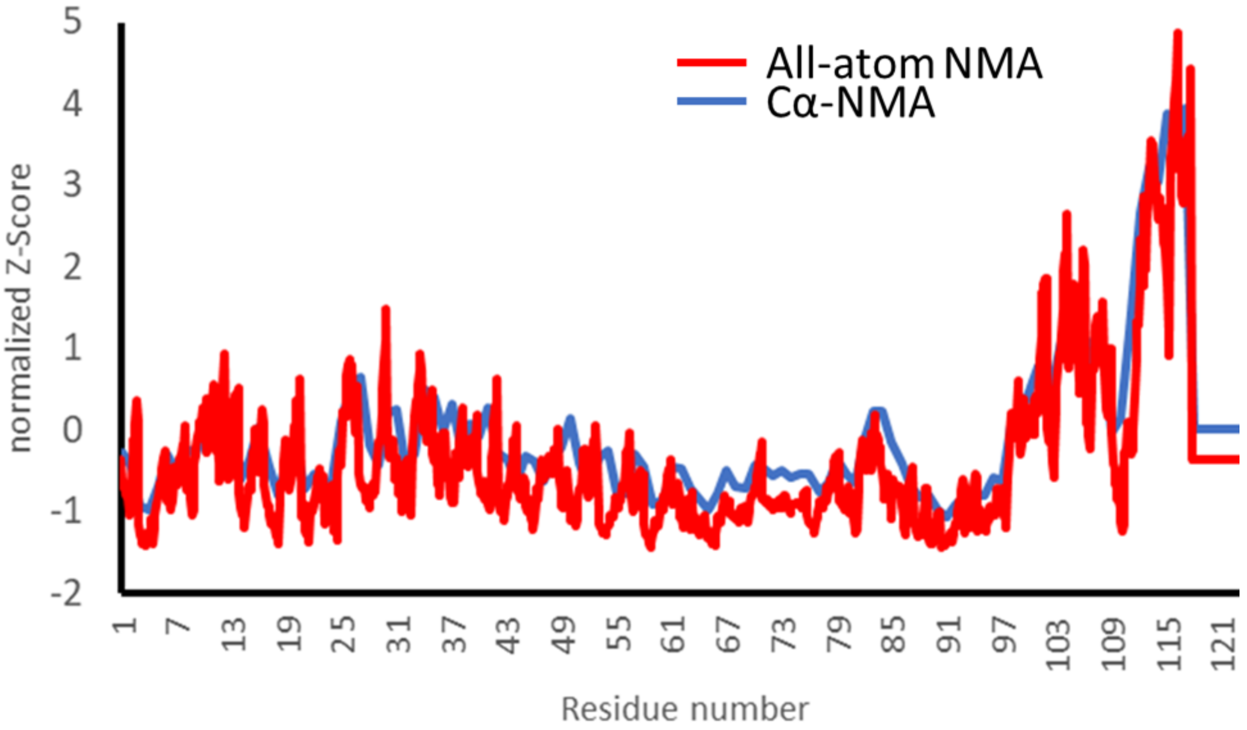

Supplementary Figure 2. Normalized Z-score comparison of all-atom NMA with Cα-NMA. The graph compares the normalized Z-scores corresponding to square fluctuation values of S13 obtained using all-atom NMA and Cα-NMA. As seen from the graph, Cα-NMA results are equivalent to all-atom NMA calculations.

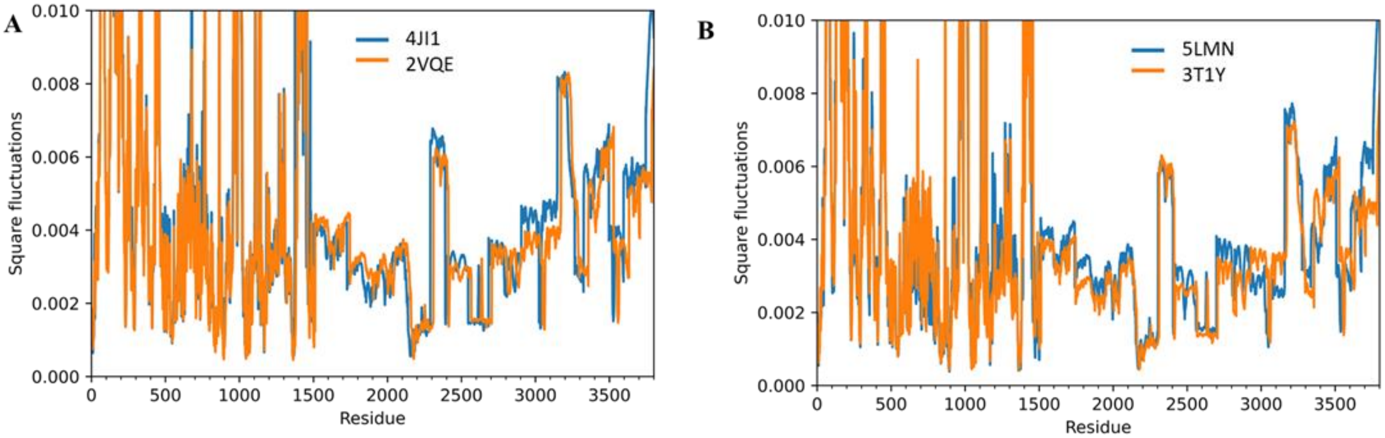

Supplementary Figure 3. Normalized square fluctuations comparison between various structures of ribosome. A. comparison of normalized square fluctuations obtained from Cα-NMA of two mRNA unbound ribosome structures with different resolutions. B. comparison of normalized square fluctuations obtained from Cα-NMA of two mRNA bound ribosome structures with different resolutions. Motions obtained using NMA remain unchanged with various structures irrespective of the resolution values

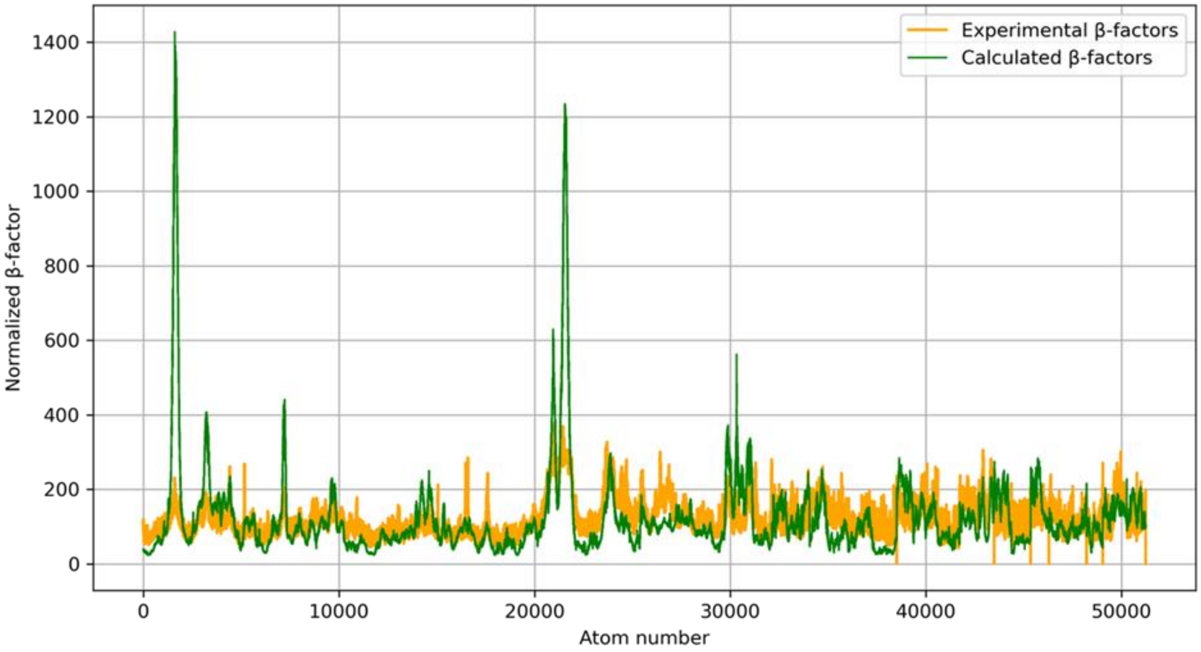

Supplementary Figure 4. Comparison between experimental and calculated b-factors. The graph here compares normalized b-factors obtained from experiment with β-factors calculated from all-atom NMA. Results imply that coarse-grained model considered for NMA analysis is comparable to the experimental values.

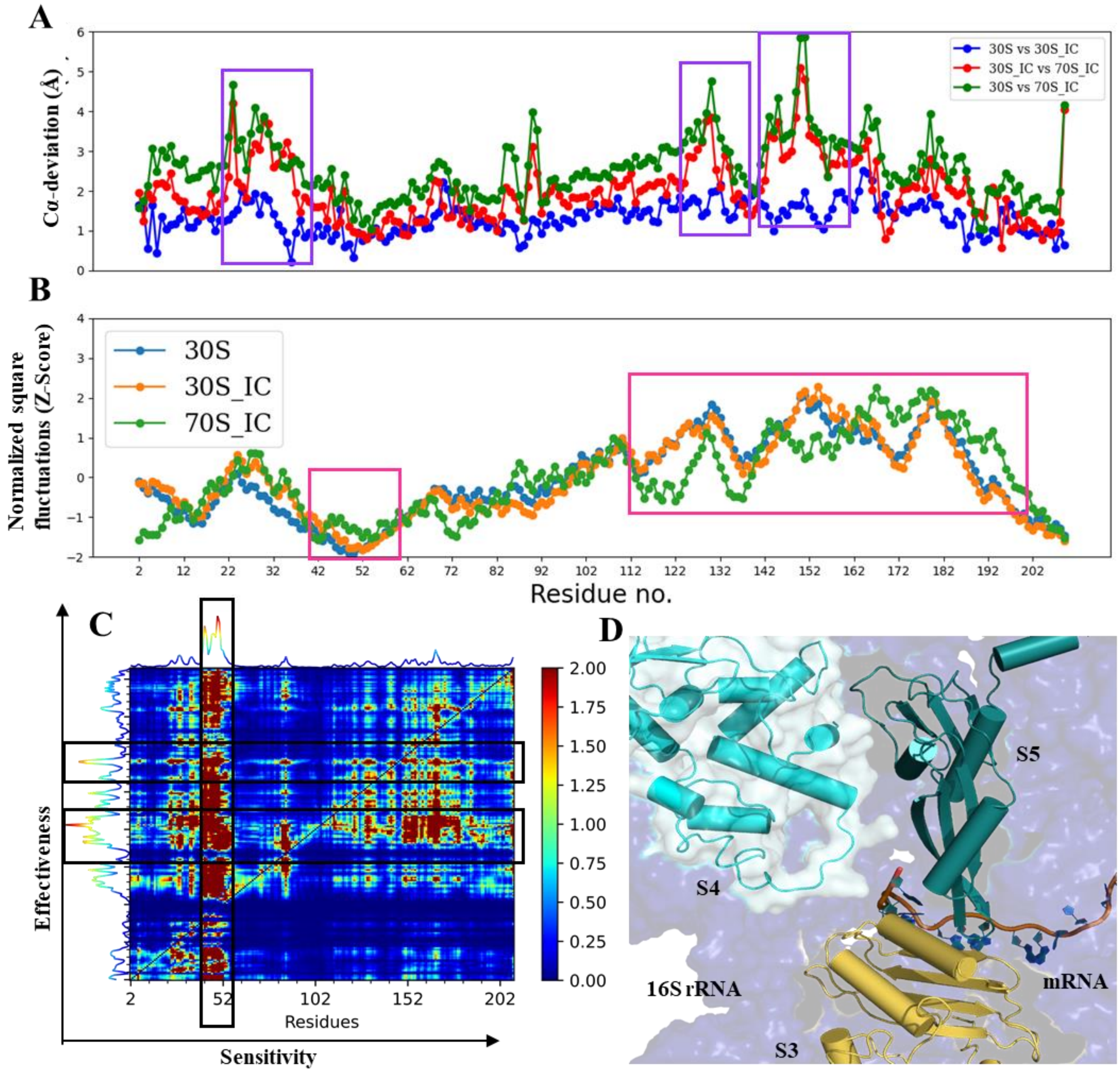

Supplementary Figure 5. Dynamics of S4

(A) Cα-deviation plot for S4 in pair wise comparison of its deviation in all the complexes is shown in a scatter plot. Higher deviation indicates higher local structural variability among those structures. The regions displaying higher local structural differences have been marked with purple rectangles.

(B) Scatter plot of normalized square fluctuations of S4 from 30S, 30S_IC and 70S_IC. Residues with Z-Score values above 2 and below -2 are considered to show higher flexibility. (in the form of

Z-Score) The regions showing significant difference in flexibility have been marked with warm pink rectangles.

(C) The PRS analysis of the S4 protein shown as a heat map with sensitivity profile on the X-axis and effectiveness profile on the Y-axis. Region marked by a vertical rectangle contain most sensitive residues while horizontal rectangle depicts the most effective residues.

(D) A region of the structural map of the 30S complex (6QNQ) highlighting the S4 (cyan) with its interacting partners, S3(yellow), S5 (teal) and mRNA in cartoon representation.

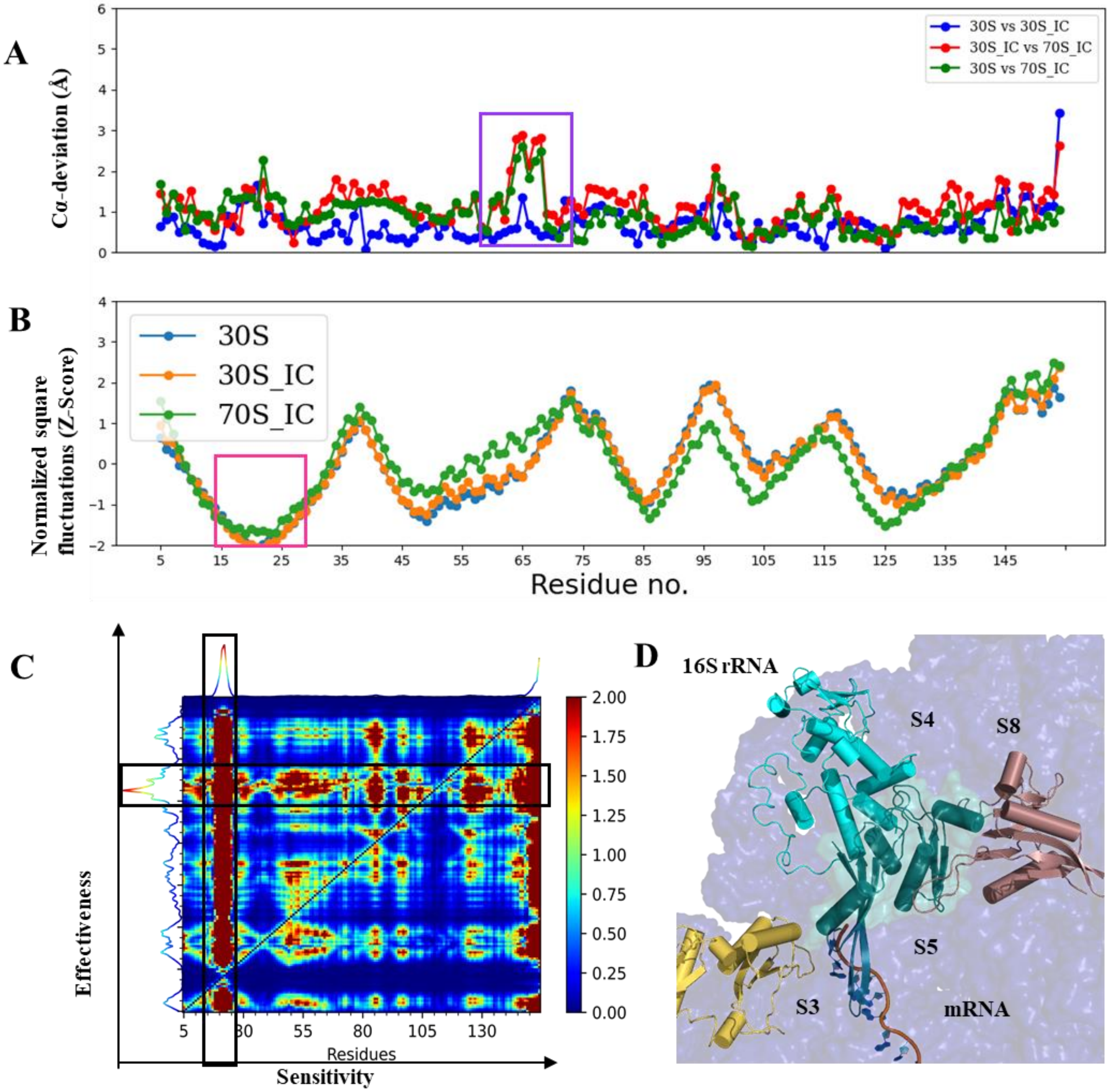

Supplementary Figure 6. Dynamics of S5

(A) Cα-deviation plot for S5 in pair wise comparison of its deviation in all the complexes is shown in a scatter plot. Higher deviation indicates higher local structural variability among those structures. The regions displaying higher local structural differences have been marked with purple rectangles.
(B) Scatter plot of normalized square fluctuations of S5 from 30S, 30S_IC and 70S_IC. (in the form of Z-Score) Residues with Z-Score values above 2 and below -2 are considered to show higher flexibility. The regions showing significant difference in flexibility have been marked with warm pink rectangles.
(C) The PRS analysis of the S5 protein shown as a heat map with sensitivity profile on the X-axis and effectiveness profile on the Y-axis. Region marked by a vertical rectangle contain most sensitive residues while horizontal rectangle depicts the most effective residues.

(D) A region of the structural map of the 30S complex (6QNQ) highlighting the S5 (teel) with its interacting partners, S3(yellow) ,S4 (cyan), S8 (brown), mRNA in cartoon and 16s rRNA in surface representation.

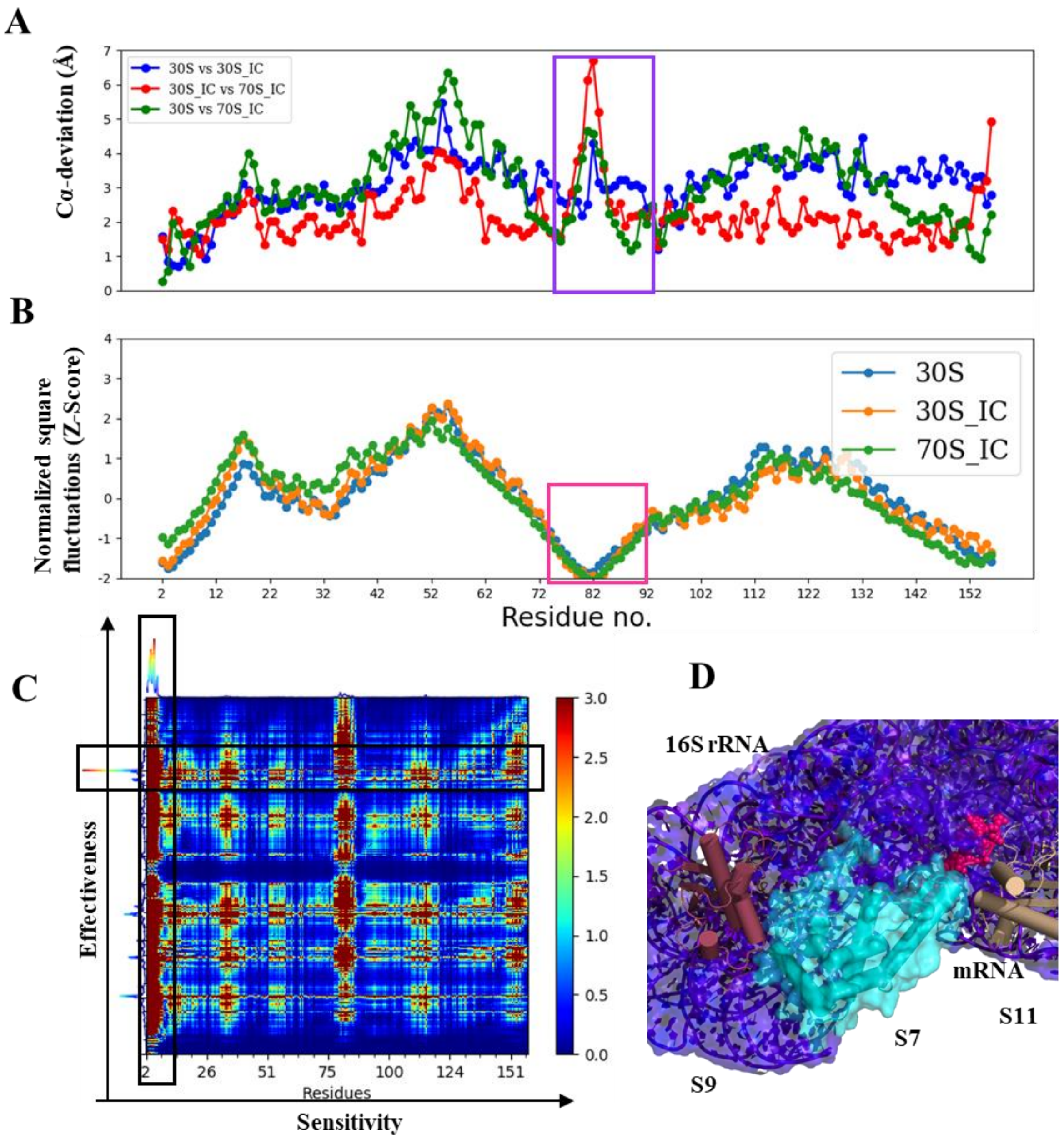

Supplementary Figure 7. Dynamics of S7

(A) Cα-deviation plot for S7 in pair wise comparison of its deviation in all the complexes is shown in a scatter plot. Higher deviation indicates higher local structural variability among those structures. The regions displaying higher local structural differences have been marked with purple rectangles.

(B) Scatter plot of normalized square fluctuations of S7 from 30S, 30S_IC and 70S_IC (in the form of Z-Score). Residues with Z-Score values above 2 and below -2 are considered to show higher flexibility. The regions showing significant difference in flexibility have been marked with warm pink rectangles.

(C) The PRS analysis of the S7 protein shown as a heat map with sensitivity profile on the X-axis and effectiveness profile on the Y-axis. Region marked by a vertical rectangle contain most sensitive residues while horizontal rectangle depicts the most effective residues.

(D) A region of the structural map of the 30S complex (6QNQ) highlighting the S7 (cyan) with its interacting partners, S11 (dark grey), S9 (brown), mRNA (pink) and 16s rRNA (blue) in surface representation.

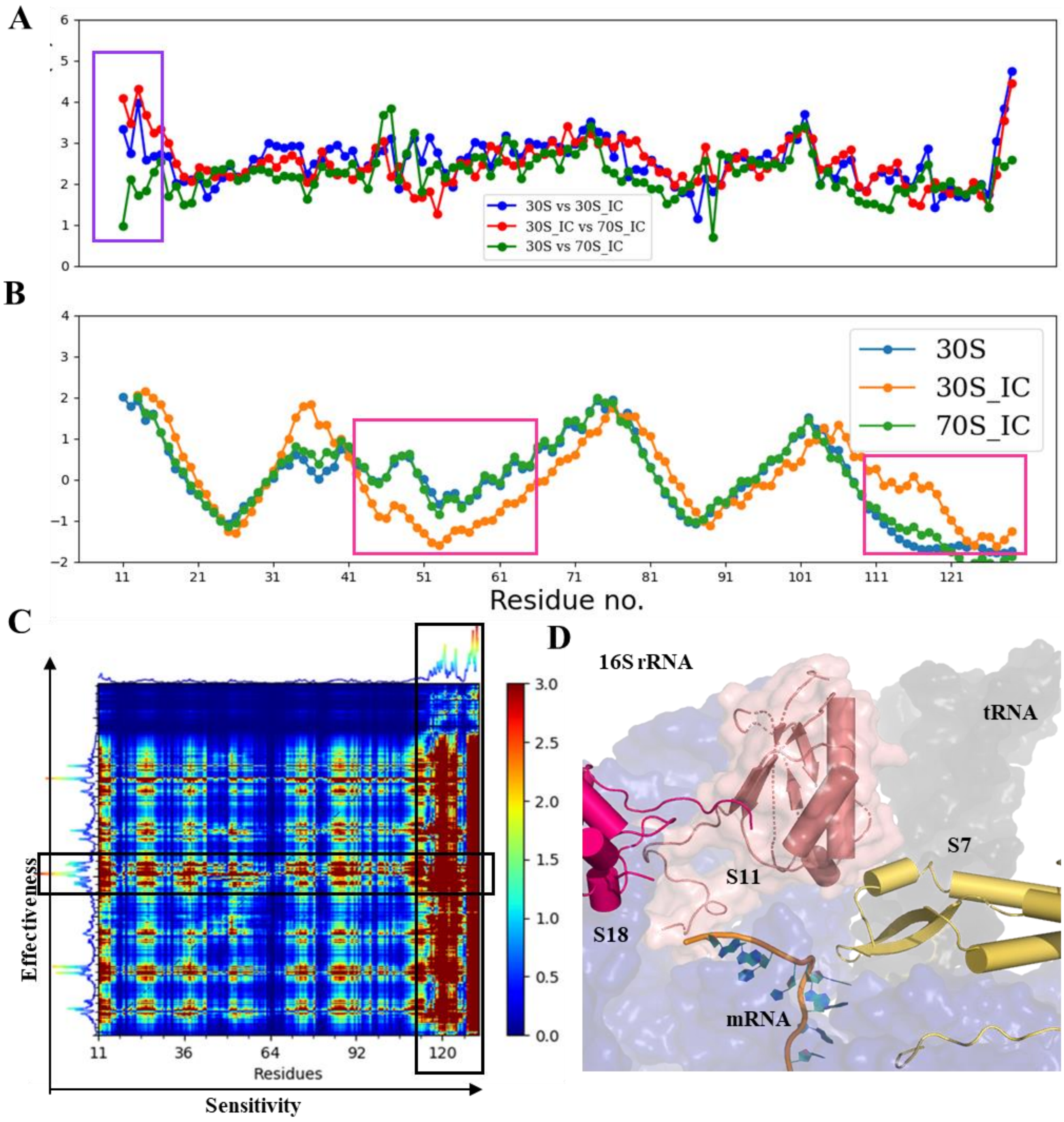

Supplementary Figure 8. Dynamics of S11

(A) Cα-deviation plot for S11 in pair wise comparison of its deviation in all the complexes is shown in a scatter plot. Higher deviation indicates higher local structural variability among those structures. The regions displaying higher local structural differences have been marked with purple rectangles.

(B) Scatter plot of normalized square fluctuations of S11 from 30S, 30S_IC and 70S_IC (in the form of Z-Score). Residues with Z-Score values above 2 and below -2 are considered to show higher

flexibility. The regions showing significant difference in flexibility have been marked with warm pink rectangles.

(C) The PRS analysis of the S11 protein shown as a heat map with sensitivity profile on the X-axis and effectiveness profile on the Y-axis. Region marked by a vertical rectangle contain most sensitive residues while horizontal rectangle depicts the most effective residues.

(D) A region of the structural map of the 30S complex (6QNQ) highlighting the S11 (salmon) with its interacting partners, S7(yellow) ,S18 (pink), tRNA (brown), mRNA in cartoon and 16s rRNA (blue) in surface representation.

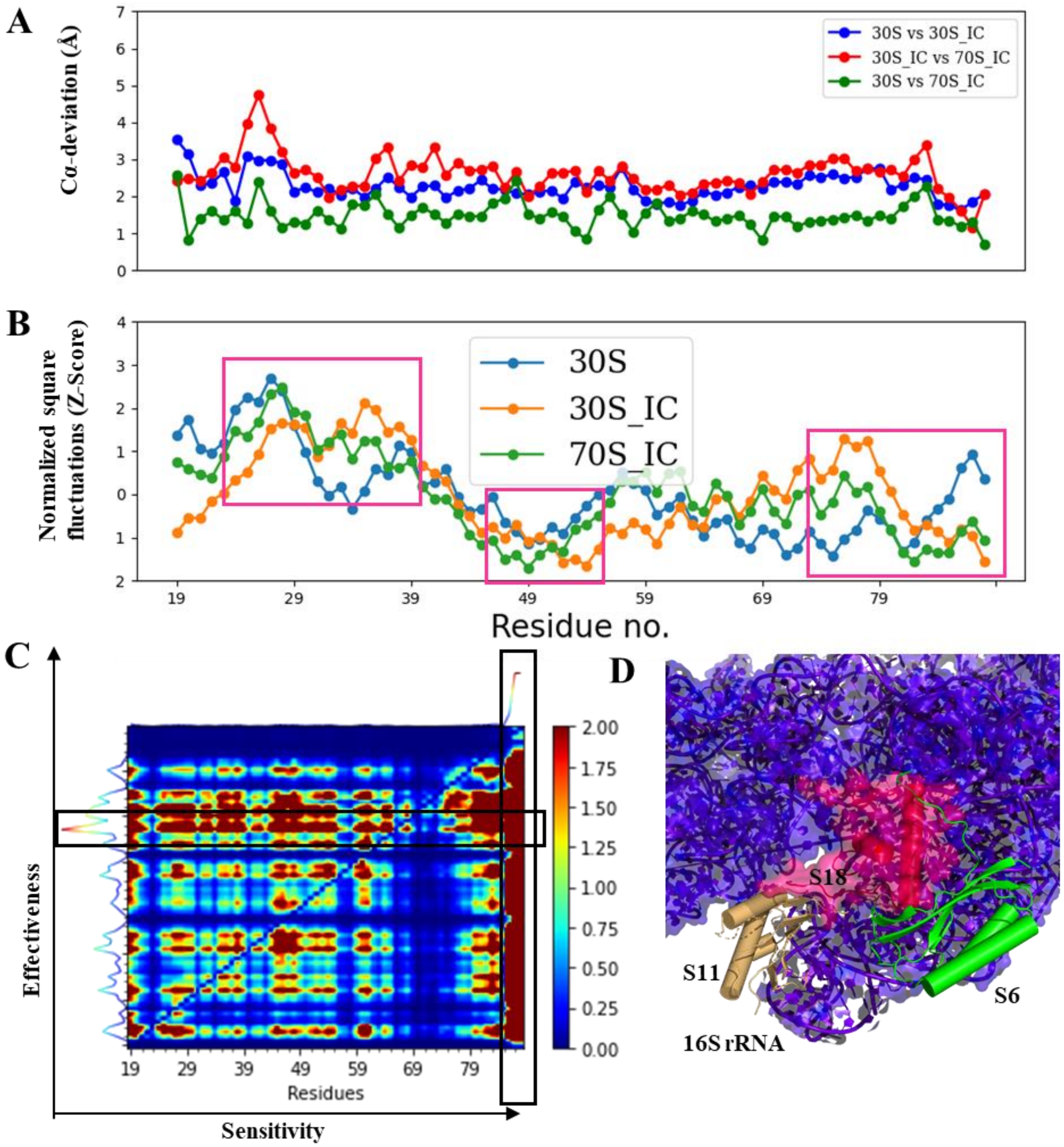

Supplementary Figure 9. Dynamics of S18

(A) Cα-deviation plot for S18 in pair wise comparison of its deviation in all the complexes is shown in a scatter plot. Higher deviation indicates higher local structural variability among those structures.

(B) Scatter plot of normalized square fluctuations of S18 from 30S, 30S_IC and 70S_IC (in the form of Z-Score). Residues with Z-Score values above 2 and below -2 are considered to show higher

flexibility. The regions showing significant difference in flexibility have been marked with warm pink rectangles.

(C) The PRS analysis of the S18 protein shown as a heat map with sensitivity profile on the X-axis and effectiveness profile on the Y-axis. Region marked by a vertical rectangle contain most sensitive residues while horizontal rectangle depicts the most effective residues.

(D) A region of the structural map of the 30S complex (6QNQ) highlighting the S18 (pink) with its interacting partners, S11(yellow), S6 (green) and 16s rRNA in surface representation.

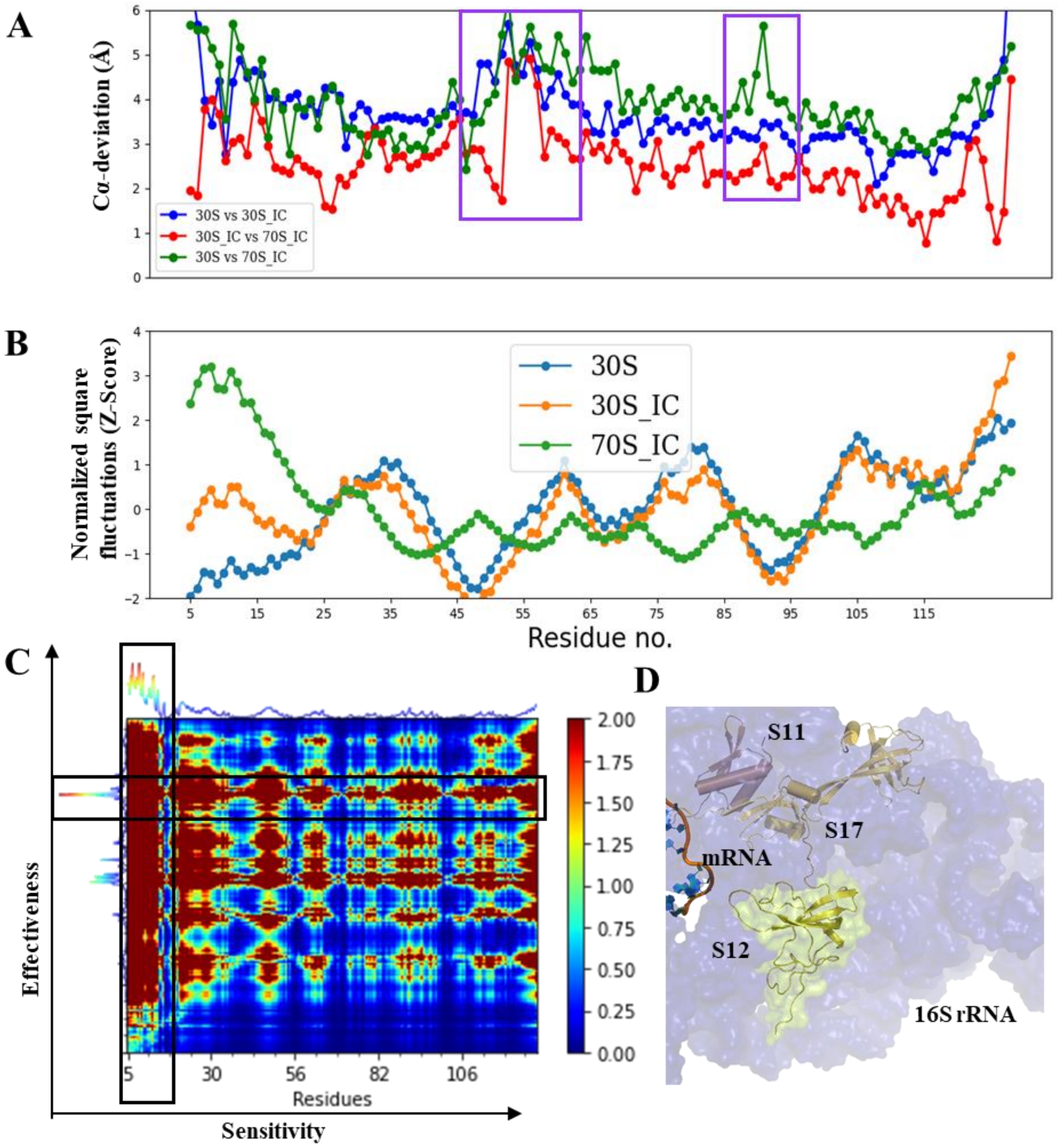

Supplementary Figure 10. Dynamics of S12

(A) Cα-deviation plot for S12 in pair wise comparison of its deviation in all the complexes is shown in a scatter plot. Higher deviation indicates higher local structural variability among those structures. The regions displaying higher local structural differences have been marked with purple rectangles.

(B) Scatter plot of normalized square fluctuations of S12 from 30S, 30S_IC and 70S_IC (in the form of Z-Score). Residues with Z-Score values above 2 and below -2 are considered to show higher flexibility.

(C) The PRS analysis of the S12 protein shown as a heat map with sensitivity profile on the X-axis and effectiveness profile on the Y-axis. Region marked by a vertical rectangle contain most sensitive residues while horizontal rectangle depicts the most effective residues.

(D) A region of the structural map of the 30S complex (6QNQ) highlighting the S12 (pale yellow) with its interacting partners, S11(brown), S17 (yellow), mRNA in cartoon and 16s rRNA (blue) in surface representation

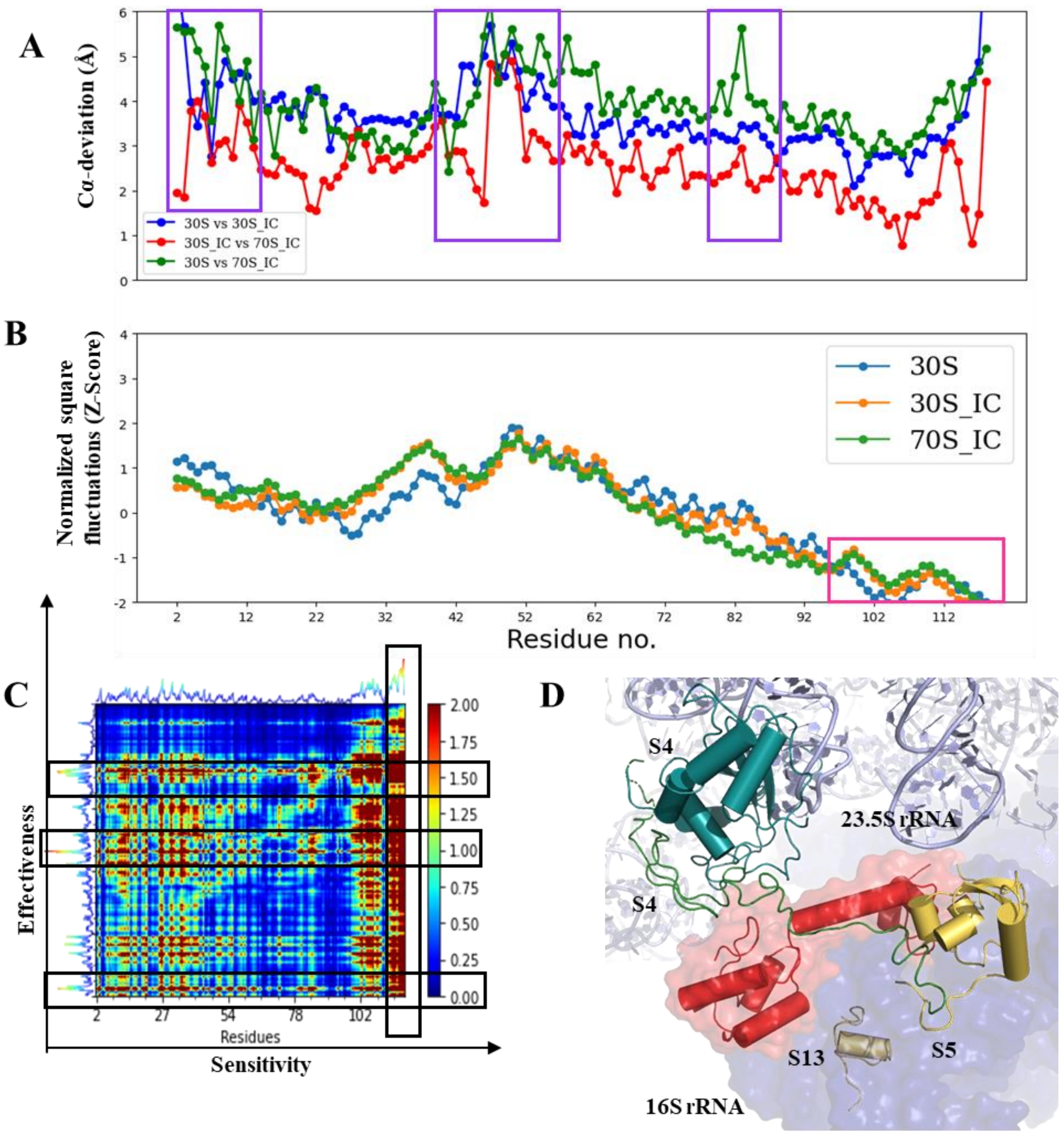

Supplementary Figure 11. Dynamics of S13

(A) Cα-deviation plot for S13 in pair wise comparison of its deviation in all the complexes is shown in a scatter plot. Higher deviation indicates higher local structural variability among those structures. The regions displaying higher local structural differences have been marked with purple rectangles.

(B) Scatter plot of normalized square fluctuations of S13 from 30S, 30S_IC and 70S_IC (in the form of Z-Score). Residues with Z-Score values above 2 and below -2 are considered to show higher

flexibility. The regions showing significant difference in flexibility have been marked with warm pink rectangles.

(C) The PRS analysis of the S13 protein shown as a heat map with sensitivity profile on the X-axis and effectiveness profile on the Y-axis. Region marked by a vertical rectangle contain most sensitive residues while horizontal rectangle depicts the most effective residues.

(D) A region of the structural map of the 30S complex (6QNQ) highlighting the S13 (red) with its interacting partners, S3(brown) ,S4 (teal), S5 (yellow), mRNA in cartoon and rRNA.

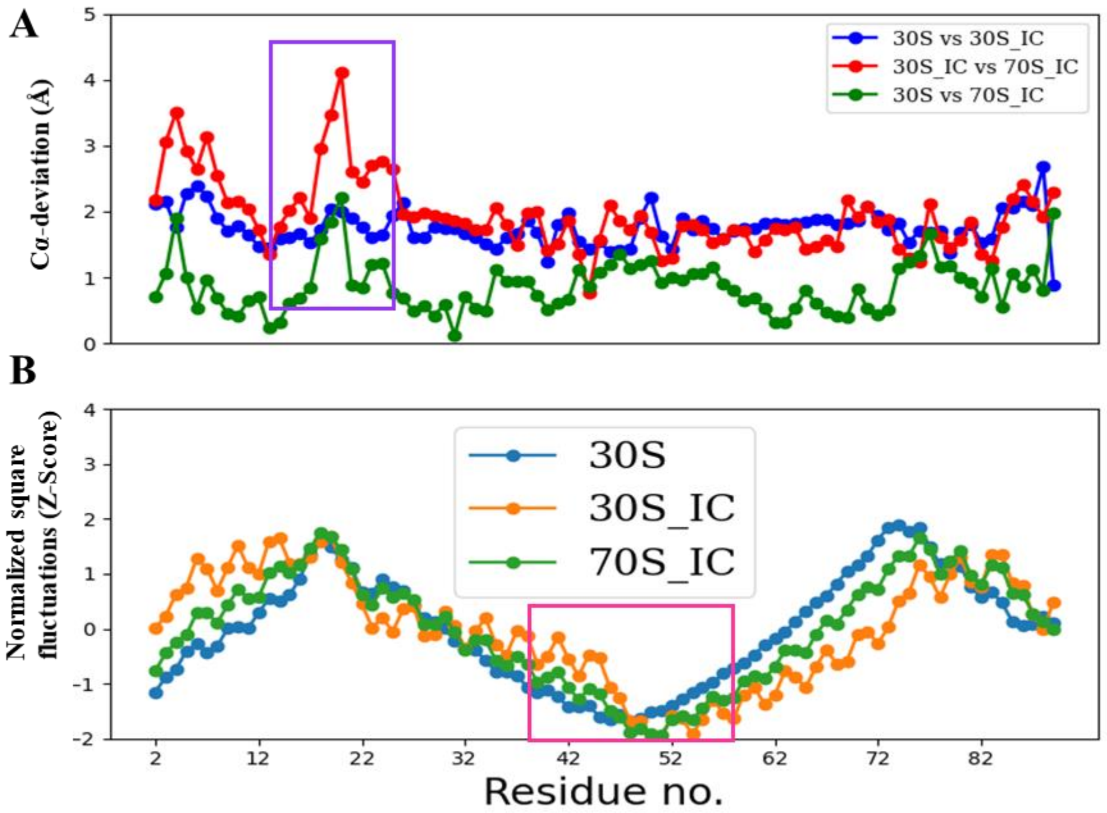

Supplementary Figure 12. Dynamics of S15

(A) Cα-deviation plot for S15 in pair wise comparison of its deviation in all the complexes is shown in a scatter plot. Higher deviation indicates higher local structural variability among those structures. The regions displaying higher local structural differences have been marked with purple rectangles.

(B) Scatter plot of normalized square fluctuations of (in the form of Z-Score) S15 from 30S, 30S_IC and 70S_IC. Residues with Z-Score values above 2 and below -2 are considered to show higher flexibility. The regions showing significant difference in flexibility have been marked with warm pink rectangles.

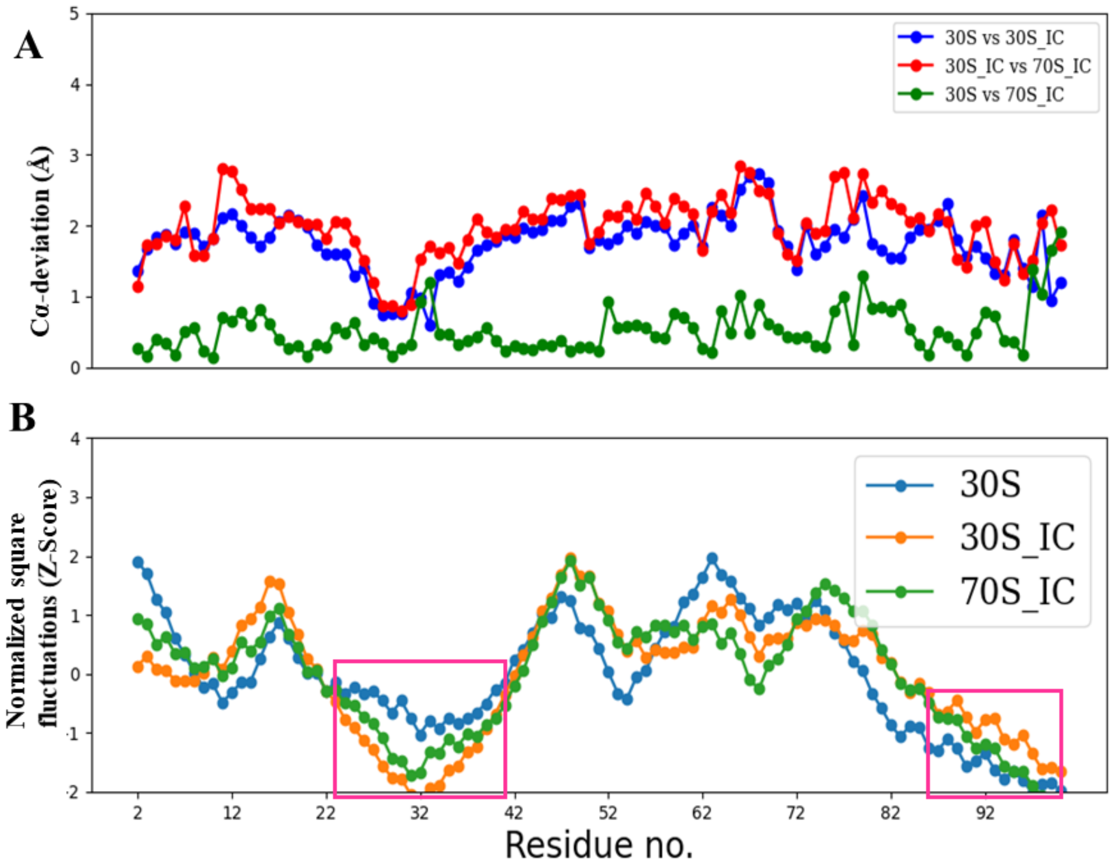

Supplementary Figure 13. Dynamics of S17

(A) Cα-deviation plot for S17 in pair wise comparison of its deviation in all the complexes is shown in a scatter plot. Higher deviation indicates higher local structural variability among those structures.

(B) Scatter plot of normalized square fluctuations of S17 from 30S, 30S_IC and 70S_IC (in the form of Z-Score). Residues with Z-Score values above 2 and below -2 are considered to show higher flexibility. The regions showing significant difference in flexibility have been marked with warm pink rectangles.

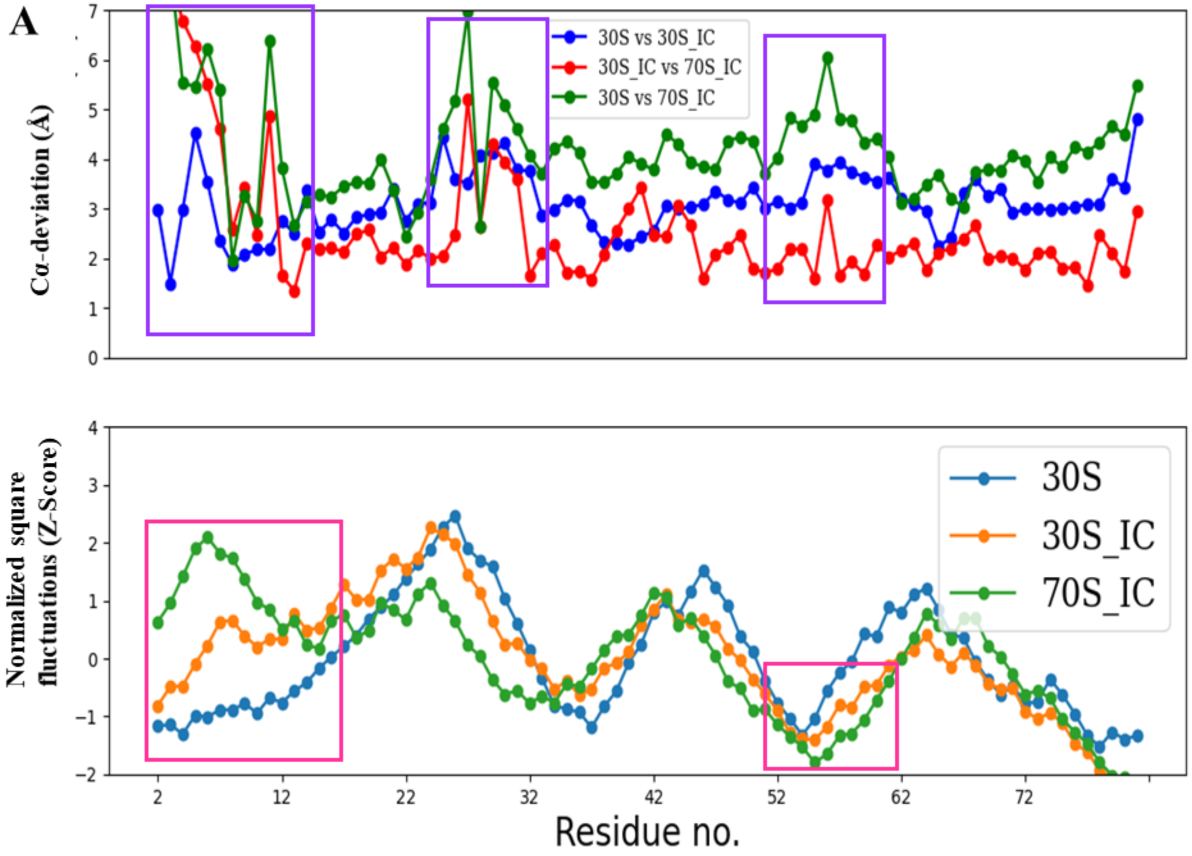

Supplementary Figure 14. Dynamics of S19

(A) Cα-deviation plot for S19 in pair wise comparison of its deviation in all the complexes is shown in a scatter plot. Higher deviation indicates higher local structural variability among those structures. The regions displaying higher local structural differences have been marked with purple rectangles.

(B) Scatter plot of normalized square fluctuations of S19 from 30S, 30S_IC and 70S_IC (in the form of Z-Score). Residues with Z-Score values above 2 and below -2 are considered to show higher flexibility. The regions showing significant difference in flexibility have been marked with warm pink rectangles.

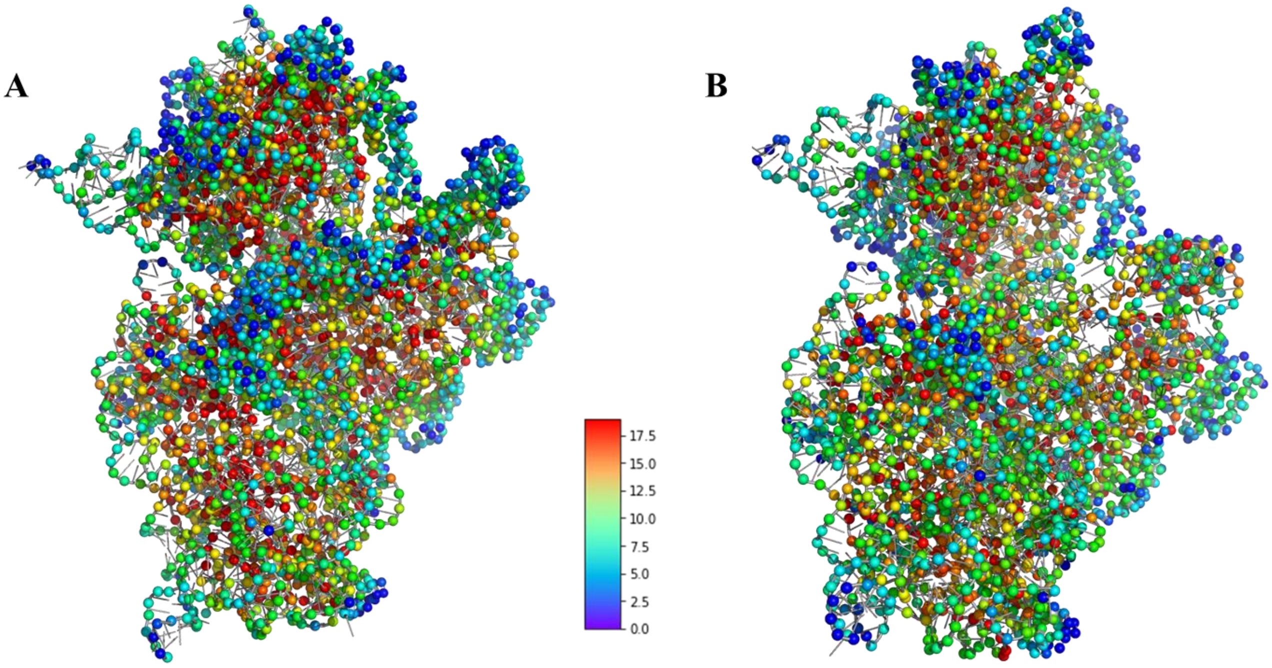

Supplementary Figure 15. Comparison of degree distribution of mRNA bound and unbound structures using residue interaction network. A. Degree distribution of mRNA unbound structure. B. Degree distribution of mRNA bound structure. From the results it is evident that binding of mRNA improves interactions among all the residues of the complex.

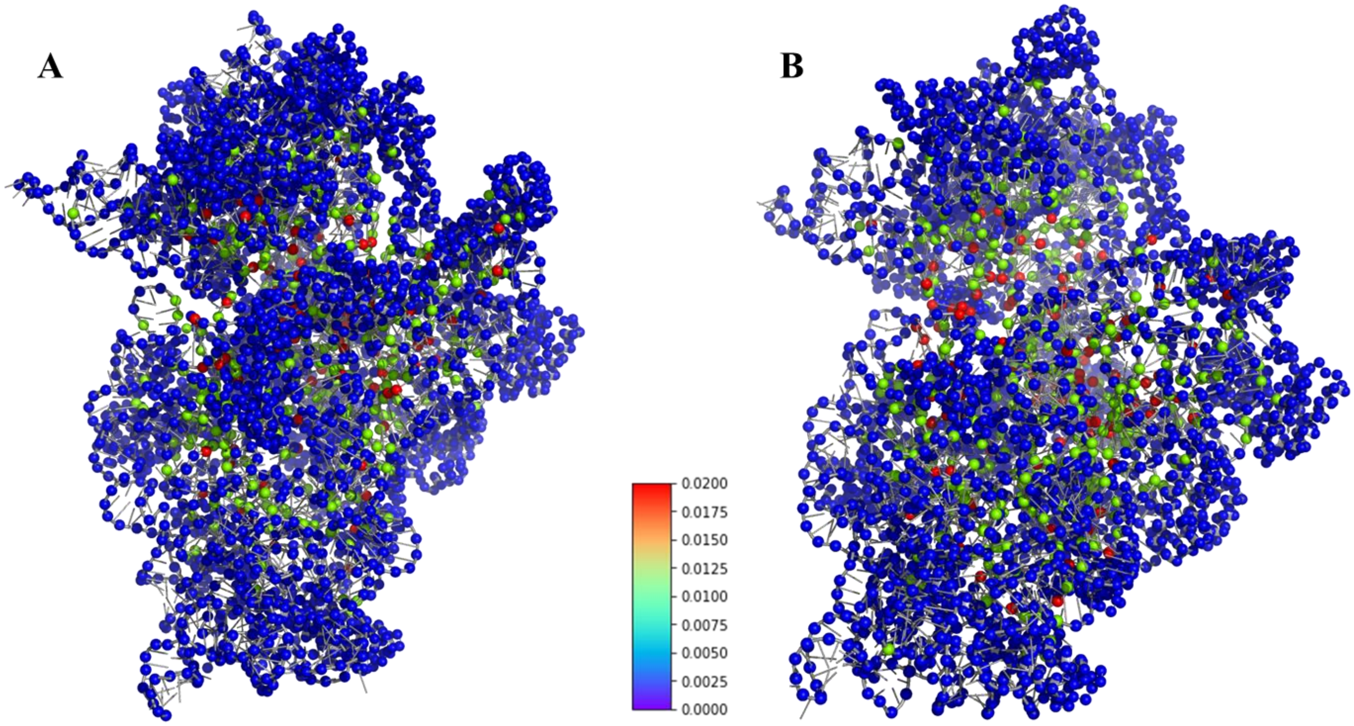

Supplementary Figure 16. Comparison of betweenness centrality of mRNA bound and unbound structures using residue interaction network. A. Betweenness distribution of mRNA unbound structure. B. Betweenness distribution of mRNA bound structure. From the results it is evident that binding of mRNA improves interactions thus improving the betweenness among all the residues of the complex.

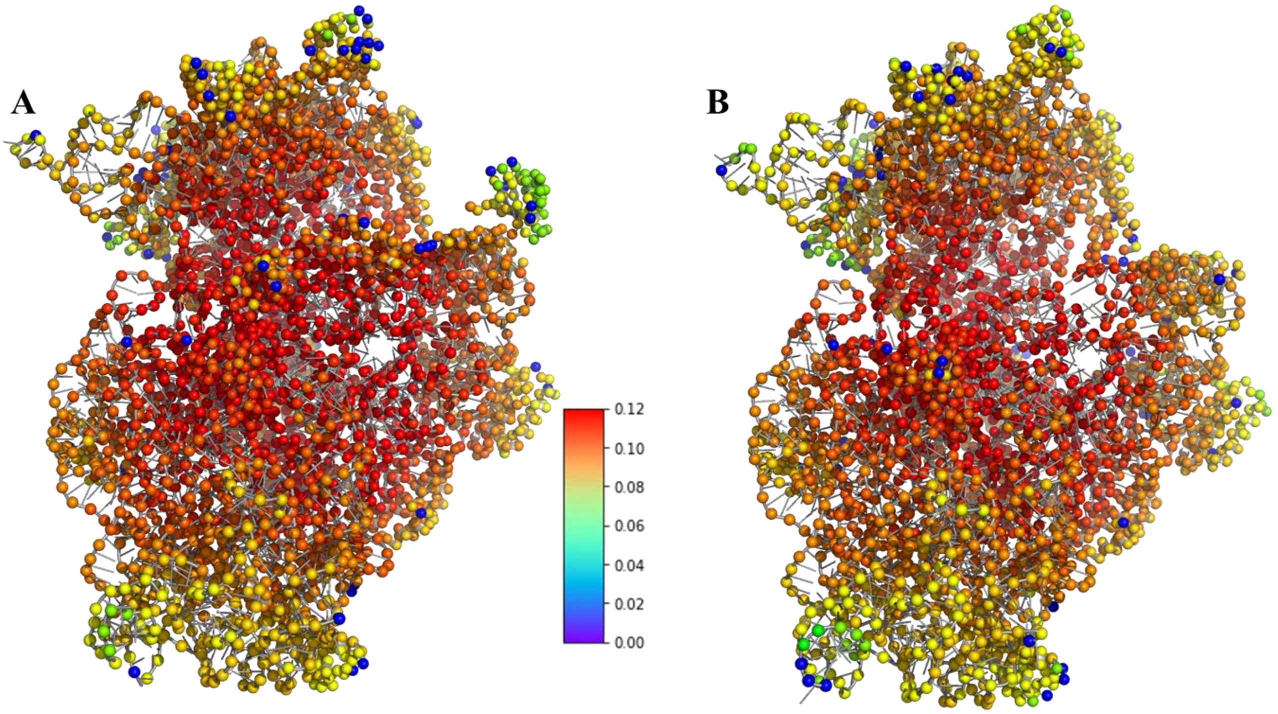

Supplementary Figure 17. Comparison of closeness centrality of mRNA bound and unbound structures using residue interaction network. A. Closeness distribution of mRNA unbound structure. B. Closeness distribution of mRNA bound structure. From the results it is evident that binding of mRNA improves interactions thus changes the interaction pathways among all the residues of the complex.

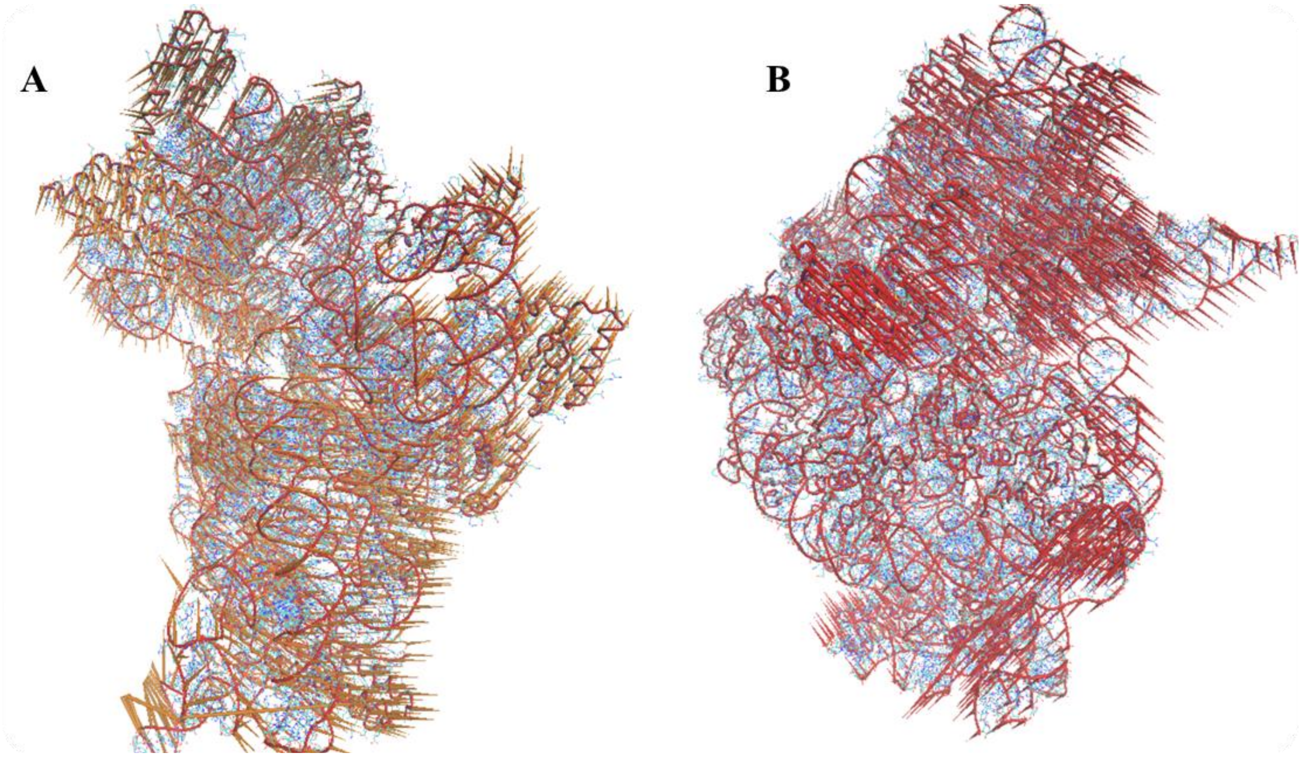

Supplementary Figure 18. Comparative normal mode analysis of (A) mRNA unbound and (B) mRNA bound ribosome structures.

3.2 Supplementary table

Supplementary table. S1. Interaction energy among various subunits of 30S complex.

Interface	ΔΔG(Kcal/mol) in 30S	ΔΔG(Kcal/mol) in 30S_IC	ΔΔG(Kcal/mol) in 70S_IC
**16srRNA-S3**	11.8494	12.5273	16.8156
**16srRNA-S4**	26.7137	36.726	45.6078
**16srRNA-S5**	4.60414	24.9667	11.1127
**16srRNA-S6**	3.78568	2.31158	0.829821
**16srRNA-S7**	18.5529	19.3527	26.3972
**16srRNA-S8**	-3.59627	-9.20682	-3.82068
**16srRNA-S9**	22.5111	38.4676	37.974
**S10-S14**	-7.02892	5.46219	-1.69678
**S11-S18**	3.667	6.66734	6.43352
**S12-S17**	-4.25619	-3.87839	-3.76717
**S13-S14**	0.0120401	0.0156266	0.0146905
**S13-S19**	0.165247	0.441622	-1.84289
**S14-S19**	-0.00904699	0.0973752	0.252362
**S15-S17**	-0.180041	-0.112854	-0.423613
**S17-S20**	0.110868	0.194394	0.0936463

**S2-S3**	0.15611	-0.014868	0.0776325
**S2-S5**	0.00475779	0.116364	0.0380321
**S2-S8**	4.36496	4.07035	3.27948
**S3-S10**	-1.1024	2.88134	2.53561
**S3-S14**	1.47286	3.30787	-1.10729
**S3-S4**	0.519433	0.320873	1.81747
**S3-S5**	0.799956	0.886873	1.03651
**S4-S16**	0.00779405	0.0491027	0.00973071
**S4-S5**	5.62564	9.9606	6.4094
**S4-S8**	0.0277013	-0.976168	-1.04953
